# The Epigenetic Factor Landscape of Developing Neocortex Is Regulated by Transcription Factors Pax6→ Tbr2→ Tbr1

**DOI:** 10.3389/fnins.2018.00571

**Published:** 2018-08-22

**Authors:** Gina E. Elsen, Francesco Bedogni, Rebecca D. Hodge, Theo K. Bammler, James W. MacDonald, Susan Lindtner, John L. R. Rubenstein, Robert F. Hevner

**Affiliations:** ^1^Center for Integrative Brain Research, Seattle Children's Research Institute, Seattle, WA, United States; ^2^Department of Environmental and Occupational Health Sciences, School of Public Health, University of Washington, Seattle, WA, United States; ^3^Nina Ireland Laboratory of Developmental Neurobiology, University of California, San Francisco, San Francisco, CA, United States; ^4^Department of Psychiatry, University of California, San Francisco, San Francisco, CA, United States; ^5^Department of Neurological Surgery, School of Medicine, University of Washington, Seattle, WA, United States

**Keywords:** cortical development, polycomb, BAF, NuRD, histone acetylation, lncRNA, microRNA, trithorax group

## Abstract

Epigenetic factors (EFs) regulate multiple aspects of cerebral cortex development, including proliferation, differentiation, laminar fate, and regional identity. The same neurodevelopmental processes are also regulated by transcription factors (TFs), notably the Pax6→ Tbr2→ Tbr1 cascade expressed sequentially in radial glial progenitors (RGPs), intermediate progenitors, and postmitotic projection neurons, respectively. Here, we studied the EF landscape and its regulation in embryonic mouse neocortex. Microarray and *in situ* hybridization assays revealed that many EF genes are expressed in specific cortical cell types, such as intermediate progenitors, or in rostrocaudal gradients. Furthermore, many EF genes are directly bound and transcriptionally regulated by Pax6, Tbr2, or Tbr1, as determined by chromatin immunoprecipitation-sequencing and gene expression analysis of TF mutant cortices. Our analysis demonstrated that Pax6, Tbr2, and Tbr1 form a direct feedforward genetic cascade, with direct feedback repression. Results also revealed that each TF regulates multiple EF genes that control DNA methylation, histone marks, chromatin remodeling, and non-coding RNA. For example, Tbr1 activates *Rybp* and *Auts2* to promote the formation of non-canonical Polycomb repressive complex 1 (PRC1). Also, Pax6, Tbr2, and Tbr1 collectively drive massive changes in the subunit isoform composition of BAF chromatin remodeling complexes during differentiation: for example, a novel switch from *Bcl7c* (Baf40c) to *Bcl7a* (Baf40a), the latter directly activated by Tbr2. Of 11 subunits predominantly in neuronal BAF, 7 were transcriptionally activated by Pax6, Tbr2, or Tbr1. Using EFs, Pax6→ Tbr2→ Tbr1 effect persistent changes of gene expression in cell lineages, to propagate features such as regional and laminar identity from progenitors to neurons.

## Introduction

Development of the embryonic cerebral cortex is regulated by intrinsic genetic programs and signaling interactions that ultimately give rise to diverse cortical areas, layers, and neuron subtypes with distinct gene expression profiles (Sun and Hevner, 2014; Silbereis et al., 2016). In each cell type, the gene expression profile is determined by a combination of transcription factors (TFs) that bind specific DNA sequences to activate or repress transcription, and epigenetic factors (EFs) that control chromatin structure and accessibility for transcription (Bernstein et al., 2007; Allis and Jenuwein, 2016). Transcriptional activity thus depends on the epigenetic status of the chromatin, as well as the presence or absence of specific TFs that bind promoters, enhancers, and other *cis*-acting regulatory elements in the genome (Nord et al., 2015; Shibata et al., 2015).

Among many important TFs in cortical development, the Pax6→ Tbr2→ Tbr1 cascade is significant because these TFs are expressed sequentially in radial glial progenitors (RGPs), intermediate progenitors (IPs), and postmitotic projection neurons (PNs), respectively (Englund et al., 2005; Hevner et al., 2006). Furthermore, these three TFs regulate important features of cortical neurons, including rostrocaudal (area) identity, PN migration, and axon projections (reviewed by Georgala et al., 2011; Mihalas and Hevner, 2017). Significantly, all three TFs are expressed in high rostral-low caudal gradients, and parallel shifts of rostrocaudal identity are found in *Pax6, Tbr2* (MGI: *Eomes*), and *Tbr1* mutant mice (Bishop et al., 2000; Bedogni et al., 2010a; Elsen et al., 2013). To explain their sequential expression, we hypothesized that Pax6, Tbr2, and Tbr1 form a genetic cascade in cortical PN lineages.

Epigenetic mechanisms are prominently involved in the etiology of intellectual disability (Iwase et al., 2017). While definitions of “epigenetics” have changed over time (Deans and Maggert, 2015; Allis and Jenuwein, 2016), most current studies recognize four broad categories of epigenetic mechanisms (Hsieh and Zhao, 2016; Yao et al., 2016): (1) DNA methylation; (2) histone covalent modifications (“marks”), such as lysine acetylation and methylation; (3) ATPase-dependent chromatin remodeling, by complexes such as BAF and NuRD; and (4) effects of non-coding RNA (ncRNA), including microRNA (miR). These epigenetic mechanisms are broadly mediated by at least 800 protein-coding EF genes, and untold numbers of ncRNA species (Medvedeva et al., 2015; Silbereis et al., 2016). In the current project, we focused on EF genes that exhibit cell-type or region-specific expression; or that are dysregulated in the neocortex of *Pax6* (Holm et al., 2007), *Tbr2* (Elsen et al., 2013; Mihalas et al., 2016), *Tbr1* (Bedogni et al., 2010a), or *Tbr1* and *Tbr2* (*Tbr1/2*; present study) mutant neocortex.

Previous studies have demonstrated physical and genetic interactions between EFs and TFs during neurogenesis. In adult subependymal zone progenitors, Pax6 forms a complex with BAF, a large, multi-subunit ATPase-dependent chromatin remodeler, to activate neurogenic genes such as *Sox11* (Ninkovic et al., 2013). In developing neocortex, Tbr2 interacts with Jmjd3 (Gene: *Kdm6b*), a histone lysine demethylase that removes repressive trimethylation marks on histone H3 lysine 27 (H3K27me3) placed by Polycomb repressive complex 2 (PRC2), to thereby derepress transcription (Sessa et al., 2017). Such interactions illustrate that TFs sometimes function by physically recruiting and targeting EFs to specific genes.

Examples where TFs and EFs regulate each other at the transcriptional level are also known. In developing forebrain, Jarid1b (*Kdm5b*), a histone lysine demethylase that removes activating epigenetic marks (H3K4me2/3) placed by Trithorax-Group (TrxG) complexes, is required to deactivate and thus limit *Pax6* expression (Albert et al., 2013). Similarly, Af9 (*Mllt3*), a YEATS domain protein that binds acetylated lysine residues, negatively modulates transcription of *Tbr1* during genesis of upper cortical layers (Büttner et al., 2010).

Conversely, Pax6, Tbr2, and Tbr1 also regulate the expression of some EF genes, although in many cases it remains unclear whether such regulation is direct. For example, *Dnmt3a* (a DNA methyltransferase) is upregulated in *Pax6* null embryonic cortex (Holm et al., 2007), but it is unknown if Pax6 regulates *Dnmt3a* directly or indirectly (Ypsilanti and Rubenstein, 2016). A few EF genes are known targets of Tbr2, such as *Gadd45g*, important in DNA demethylation (Sessa et al., 2017). Tbr1 is known to activate *Auts2* (Bedogni et al., 2010a), a Polycomb repressive complex 1 (PRC1) non-canonical subunit (Gao et al., 2014); and *Arid1b*, an important BAF subunit (Notwell et al., 2016). Building on these few examples, one goal of the present study was to comprehensively identity EF genes that are directly bound and regulated by Pax6, Tbr2, and Tbr1.

In addition to studying regulation of EF genes, we also wished to characterize EF genes associated with cortical differentiation, comprising the “EF landscape.” In embryonic neocortex, histological zones are correlated with cell identity and differentiation (Bystron et al., 2008), while rostrocaudal and mediolateral gradients of gene expression presage arealization (O'Leary et al., 2007). Indeed, zonal expression patterns can be used to infer specificity of gene expression in RGPs, apical IPs, basal IPs, and neurons (Kawaguchi et al., 2008). In the present study, by combining microarray analysis of RGP and IP transcriptomes (Nelson et al., 2013) with *in situ* hybridization (ISH) to define gene expression patterns, we find that dozens of EF genes exhibit cell-type or region-specific expression, and together constitute a rich EF landscape involving all categories of epigenetic mechanisms.

Our analysis depicts a new, comprehensive view of the EF landscape in developing neocortex, and its regulation by Pax6, Tbr2, and Tbr1. In addition, this approach yields an updated portrayal of the Pax6→ Tbr2→ Tbr1 cascade, including feedforward and feedback regulation. Importantly, the data indicate that Pax6 is not a specific marker of RGPs, but is also expressed in many Tbr2+ IPs, as we have noted (Englund et al., 2005). Other TFs, such as Sox9, are more specific RGP markers. Together, our results show how a cortical TF network implements cortical differentiation by controlling diverse EFs.

## Materials and methods

### Data sources

To study gene expression and regulation in the context of cortical neurogenesis, we analyzed data from experiments using embryonic mouse cortex, in the age range from embryonic day (E) 13.5 to E15.5. For microarray and chromatin immunoprecipitation-sequencing (ChIP-seq) experiments, data were reanalyzed from previous studies, and from a new microarray dataset (Supplementary Table S1). For *in situ* hybridization (ISH), data were sourced from Genepaint (http://genepaint.org); the Allen Brain Atlas Developing Mouse Brain (http://developingmouse.brain-map.org/); the Brain Gene Expression Map (BGEM), hosted at Gensat (http://gensat.org); and previous literature.

### Screen to identify cell-type and region-specific gene expression

Previously, transcriptome profiling and unbiased cluster analysis of single cells indicated that the ventricular zone (VZ) and subventricular zone (SVZ) of E14.5 mouse neocortex contain four cell types: RGPs, apical IPs (aIPs), basal IPs (bIPs), and postmitotic projection neurons (PNs) (Kawaguchi et al., 2008). Each cell type occupies characteristic histological zones in developing neocortex: RGPs in VZ; aIPs in VZ; bIPs in SVZ; and PNs in SVZ, intermediate zone (IZ), and cortical plate (CP). Using this information, we screened the top 300 differentially expressed genes (up- and downregulated) from a previous microarray experiment comparing RGP and IP transcriptomes (Nelson et al., 2013). For the selected genes, we assessed histological expression patterns as revealed by ISH or microdissection (Ayoub et al., 2011). The primary goal was to identify RGP and IP genes, but as it happened, PN-specific genes were also enriched in *Tbr2*-GFP+ sorted cells, reflecting perdurance of GFP in daughter neurons of IPs (Nelson et al., 2013). Conversely, non-PN lineages (e.g., meninges) were highly enriched in *Tbr2*-GFP^−^ sorted cells.

Cell-type specificity was determined using the following criteria. RGP genes were enriched in *Tbr2*-GFP^−^ cells on microarray (log_2_FC < 0; *p* < 0.05), and expressed mainly in VZ; aIP genes were enriched in *Tbr2*-GFP+ cells (log_2_FC > 0; *p* < 0.05), and expressed mainly in VZ; bIP genes were enriched in *Tbr2*-GFP+ cells, and expressed mainly in SVZ; PN genes were enriched in *Tbr2*-GFP+ cells, and expressed in IZ/CP. Some neuronal differentiation genes were expressed by not only neurons, but also progenitor cells undergoing neuronal differentiation. Also, some neuronal genes were widely expressed in forebrain neurons, while others were restricted to cortical PNs. Thus, neuron-specific genes were further classified according to initial zone of expression (VZ earliest, CP latest), and specificity for cortical or general neurons. If different microarray probes for the same gene showed enrichment in *Tbr2*-GFP+ and *Tbr2*-GFP^−^ cells (“conflicted” probes), the gene was not considered specific for cell type. Genes with rostrocaudal expression gradients were identified, and classified according to zone of expression, as previously described (Bedogni et al., 2010a; Elsen et al., 2013; Alfano et al., 2014). Further details of our approach, including analysis of gene expression in other cell types (such as GABAergic neurons), will be presented in a separate manuscript (in preparation).

By this approach, 52 EF genes with cell-type-specific expression in developing neocortex were ascertained (Supplementary Table S2), as were 11 EF genes with rostrocaudal gradients; 4 genes exhibited both cell-type and region-specific expression (Supplementary Table S3).

### New microarray analyses of *Tbr1, Tbr2*, and *Tbr1/2* deficient cortex

*Tbr1* knockout (KO), *Tbr2* conditional knockout (cKO), and *Tbr1/2* double KO/cKO (dKO) mouse embryos were produced as described (Bedogni et al., 2010a; Elsen et al., 2013). The *Tbr1/2* double mutants were generated by breeding to combine the necessary alleles (*Tbr1*^−/−−^*;Tbr2*^2*F*/2*F*^*;Nes11*^*Cre*^). On E14.5, embryos were harvested, and neocortex was immediately dissected and frozen as described (Elsen et al., 2013). Genotypes were determined by PCR of tail DNA. Controls were wild type (+/+) for Tbr1 and non-recombined for *Tbr2*. RNA was purified from neocortex, quality checked, and submitted for microarray analysis (Affymetrix Mouse Exon 1.0 ST). Each embryonic neocortex was an independent biological replicate. The number of samples (*n*) of each genotype was: 3 control, 4 *Tbr1* KO, 2 *Tbr2* cKO, and 3 *Tbr1/2* dKO. The microarray results were analyzed statistically as described (Elsen et al., 2013). In the current paper, we also analyzed previous microarray data from *Tbr1* KO (Bedogni et al., 2010a) and *Tbr2* cKO (Elsen et al., 2013) neocortices, designated microarray 1 (MA1); the new microarray data were designated microarray 2 (MA2). *Tbr1/2* dKO neocortex was analyzed only in MA2 (Supplementary Table S1). The new microarray data reported in this paper have been deposited in the Gene Expression Omnibus (GEO) database, www.ncbi.nlm.nih.gov/geo (accession no. GSE115703).

### Ethics statement

This study was carried out in accordance with the recommendations of Guide for the Care and Use of Laboratory Animals, National Research Council. The protocol was approved by the Institutional Animal Care and Use Committee of Seattle Children's Research Institute.

### Analysis of ChIP-Seq and other TF binding data

Previous ChIP-seq raw data were obtained and reanalyzed for Pax6 (Pattabiraman et al., 2014), Tbr2 (Sessa et al., 2017), and Tbr1 (Notwell et al., 2016). TF binding sites (peaks) were determined from BED files using the Bioconductor ChIPpeakAnno package (Zhu et al., 2010), as well as the TxDb.Mmusculus.UCSC.mm9.knownGene package, which is simply a re-packaging of the UCSC known gene table for the mm9 genome build (Rosenbloom et al., 2015). Peaks were annotated to the closest gene within 50 kilobases (kb) of the binding site. In the present analysis, TF binding was considered “positive” if the binding site was located anywhere in the transcribed sequence, or within 50 kb upstream or downstream.

The ChIP-seq data listed in Supplementary Table S1 were our main sources, but TF binding was also evaluated by reference to previous literature. For Pax6, previous studies included genome-wide ChIP analyses of Pax6 binding in E12.5 neocortex (Sansom et al., 2009) and forebrain (Sun et al., 2015); as well as computational analysis and prediction of Pax6 binding sites (Coutinho et al., 2011). Results of all TF binding analyses for selected EF genes are included in Supplementary Table S3.

### Defining direct target genes regulated by transcription factors

Genes were defined as direct targets of Pax6, Tbr2, or Tbr1 regulation if the gene showed both TF binding by ChIP-seq, and differential expression (*p* < 0.05) in TF mutant neocortex compared to control on microarray. For analysis of Tbr1 and Tbr2 direct target genes, differential expression (*p* < 0.05) on either MA1 or MA2 was accepted as evidence of regulation. Genes regulated synergistically by Tbr1 and Tbr2 were identified by the presence of both Tbr1 and Tbr2 binding sites, and significant differential expression (*p* < 0.05) in *Tbr1/2* dKO cortex, but not in *Tbr1* KO or *Tbr2* cKO cortex independently.

By this approach, 36 EF genes were identified as direct targets of transcriptional regulation by Pax6, Tbr2, and/or Tbr1; direct regulation was also assessed for the key TFs Pax6, Insm1, Tbr2, and Tbr1 (Supplementary Table S4).

## Results and discussion

### Cell-type specific expression of *Pax6, Tbr2*, and *Tbr1*

**Figure 1:**
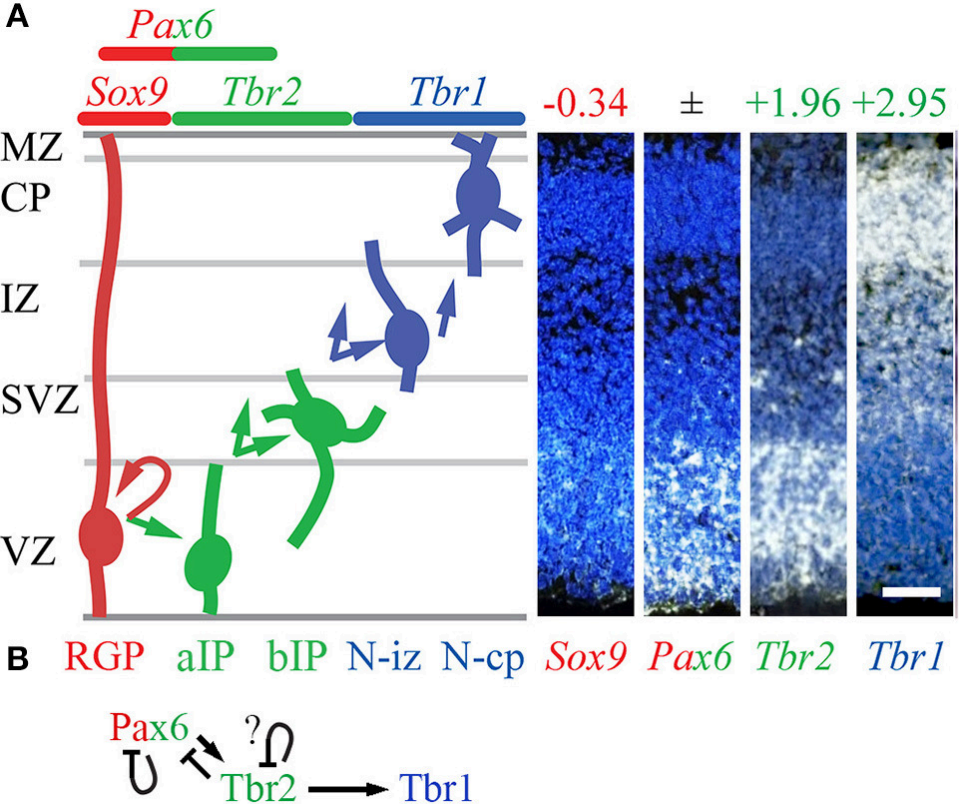
Cell types, TF expression, and histological zones in E14.5 mouse neocortex. **(A)** Neurogenesis and cell-type-specific TF expression. Histological zones and cell types (left) are aligned with TF gene ISH (right; white ISH signal, blue nuclear counterstain). Arrows indicate common (but not exclusive) pathways of neurogenesis. Numbers above ISH panels indicate log_2_FC on *Tbr2*-GFP microarray (all *p* < 0.05). Abbreviations: see text. ISH: Allen Brain Atlas Developing Mouse Brain, E15.5 (colors inverted for figure). Scale bar: 50 μm. (**B**) The Pax6→ Tbr2→ Tbr1 cascade involves direct feedforward activation (arrows) and feedback repression (bars). The effect of Tbr2 binding at the *Tbr2* locus could not be determined from available data (see text), but could be feedback repression.

Using the methods described above to evaluate cell-type-specific gene expression, we began by evaluating the expression of *Pax6, Tbr2, Tbr1*, and other selected TFs. As expected, *Tbr2* and *Tbr1* were highly enriched in the *Tbr2*-GFP+ lineage, and showed zonal expression patterns on ISH consistent with IPs (aIPs and bIPs) and PNs, respectively (Figure 1A). However, *Pax6* expression was not cell-type-specific: different probes for *Pax6* on the *Tbr2*-GFP microarray were enriched in different cell groups (conflicted probes), while ISH showed *Pax6* in both VZ and SVZ (Figure 1A). These results accord with our previous observations that Pax6 protein is expressed not only in RGPs, but also in some IPs (Englund et al., 2005). However, other TFs were identified as specific markers of RGPs, such as *Sox9* (Figure 1A). Immunohistochemistry and genetic lineage tracing have confirmed that *Sox9* is specifically expressed in RGPs (Kaplan et al., 2017).

### Feedforward and feedback regulation in the Pax6→ Tbr2→ Tbr1 cascade

Using an intersectional approach to identify genes that were both bound and regulated by each TF (details in section Materials and Methods), we first examined whether Pax6, Tbr2, and Tbr1 transcriptionally regulate each other and/or themselves.

Previous studies have found that Pax6 directly represses its own transcription (Manuel et al., 2007), and directly activates *Tbr2* expression (Sansom et al., 2009). Our analysis confirmed that both *Pax6* and *Tbr2* were bound and regulated by Pax6. In *Pax6* null (*Pax6*^*Sey*/*Sey*^) neocortex, expression of *Pax6* (non-functional mRNA) was greatly increased (log_2_FC = +1.20; *p* = 10^−6^), while *Tbr2* was greatly decreased (log_2_FC = −1.07; *p* = 10^−6^).

Previous studies have also suggested that Tbr2 directly binds and activates *Tbr1* (Sessa et al., 2017). This was confirmed in the present analysis. Moreover, we found that Tbr2 binds and represses *Pax6*: in *Tbr2* cKO neocortex, *Pax6* was significantly upregulated (log_2_FC = +0.36, *p* = 10^−3^ on MA1; log_2_FC = +0.49, *p* = 10^−3^ on MA2). In contrast, *Tbr1* was downregulated in *Tbr2* cKO cortex. We also noted Tbr2 binding to its own gene (*Tbr2*), although the functional effects were uncertain: *Tbr2* mRNA expression is reduced due to *Tbr2* cKO (Elsen et al., 2013), so the effects of Tbr2 on its own transcription could not be evaluated. We speculate that, like Pax6, Tbr2 may repress its own transcription as a feedback mechanism (Figure 1B).

ChIP-seq analysis of Tbr1 showed that Tbr1 binds to the *Tbr2* locus, but not to *Pax6* or *Tbr1*. On microarray, however, *Tbr2* expression was not significantly changed in *Tbr1* null mice (S3). Thus, Tbr1 does not appear to directly regulate *Tbr2, Pax6*, or *Tbr1*.

Together, these data indicate that the Pax6→ Tbr2→ Tbr1 cascade operates as a positive feedforward cascade, but also self-regulates by direct negative feedback effects (Figure 1B).

Since Pax6, Tbr2, and Tbr1 are expressed in different cell types (differentiation stages in the same lineage)—except for overlapping expression of Pax6 and Tbr2 in some IPs (Englund et al., 2005)—feedforward activation may involve epigenetic mechanisms. For example, Tbr2 and Tbr1 exhibit virtually no overlap of protein expression in developing neocortex, yet Tbr2 expression in IPs is essential for high levels of Tbr1 expression in postmitotic PNs (S4). One explanation is that Tbr2 may drive epigenetic changes at the *Tbr1* locus that persist in postmitotic neurons. For example, removal of repressive histone marks by Jmjd3, an interacting protein of Tbr2, may create a permissive chromatin environment for *Tbr1* transcription (Sessa et al., 2017).

### Identification of EFs with cellular, regional, or TF-regulated expression

To identify genes with cell-type or region-specific expression in E14.5 mouse neocortex, we screened differentially expressed genes from a previous microarray experiment comparing RGP and IP transcriptomes (Nelson et al., 2013). We used ISH to characterize expression patterns in developing neocortex (Supplementary Figure S1; Section Materials and Methods). To identify EF genes regulated by Pax6, Tbr2, and Tbr1, we selected EF genes that were both bound by the TF per ChIP-seq, and significantly regulated in TF null neocortex per microarray. All EF genes that were evaluated are listed in Supplementary Table S3, which also includes results from microarrays, ISH, and ChIP-seq; annotations of cell-type and regional identity; and previous literature citations.

Of more than 350 EF genes evaluated, 52 exhibited cell-type-specific expression: 14 in RGPs, 2 in aIPs, 6 in bIPs, 9 in aIPs and bIPs, 18 in general neurons or precursors, and 3 in PNs or precursors (Supplementary Table S2). In addition, 11 EF genes exhibited rostrocaudal gradients: 4 high rostral, 7 high caudal (Supplementary Table S3). Furthermore, 36 EF genes were bound and regulated by Pax6, Tbr2, and/or Tbr1 (Supplementary Table S4). Of these, 9 were regulated by two TFs independently, but always in the same direction; and 2 EF genes were regulated only synergistically by Tbr2 and Tbr1. The effects of TFs on target gene expression were mixed: Pax6 activated 5 EF genes, and repressed 5; Tbr2 activated 8, and repressed 10; Tbr1 activated 13, and repressed 2; Tbr1 and Tbr2 (Tbr1/2) coordinately activated 2 EF genes. In sum, 73 EF genes showed cell-type or regional specificity, or were directly regulated by at least one of the TFs (Pax6, Tbr2, and Tbr1).

Results for each category of EFs are presented and discussed in the following sections. Neurodevelopmental implications are discussed in the final sections.

### DNA methylation and demethylation

DNA methylation (5-methylcytosine on CpG) mediates chromatin compaction and gene silencing, and is actively regulated during neurogenesis (Moore et al., 2013; Sharma et al., 2016). DNA methylation is mediated by N-methyltransferases (*Dnmt1/3a/3b*), and can be reversed (erased) by pathways involving *Gadd45a/b/g, Tet*, and *Aicda* genes (Moore et al., 2013; Matsunaga et al., 2015). Dnmt1 is active on hemimethylated DNA in newly replicated cells, while Dnmt3a/3b catalyze targeted *de novo* methylation. Silencing of methylated DNA is mediated by “reader” proteins, such as methyl-binding domain proteins (*Mecp2* and *Mbd* genes), and zinc-finger proteins such as Kaiso (*Zbtb33*), Zbtb4, and Zbtb38. Dnmt activity can also be modulated by factors such as Np95 (*Uhrf1*), a histone reader that stabilizes and potentiates Dnmt1 (Murao et al., 2014).

**Figure 2:**
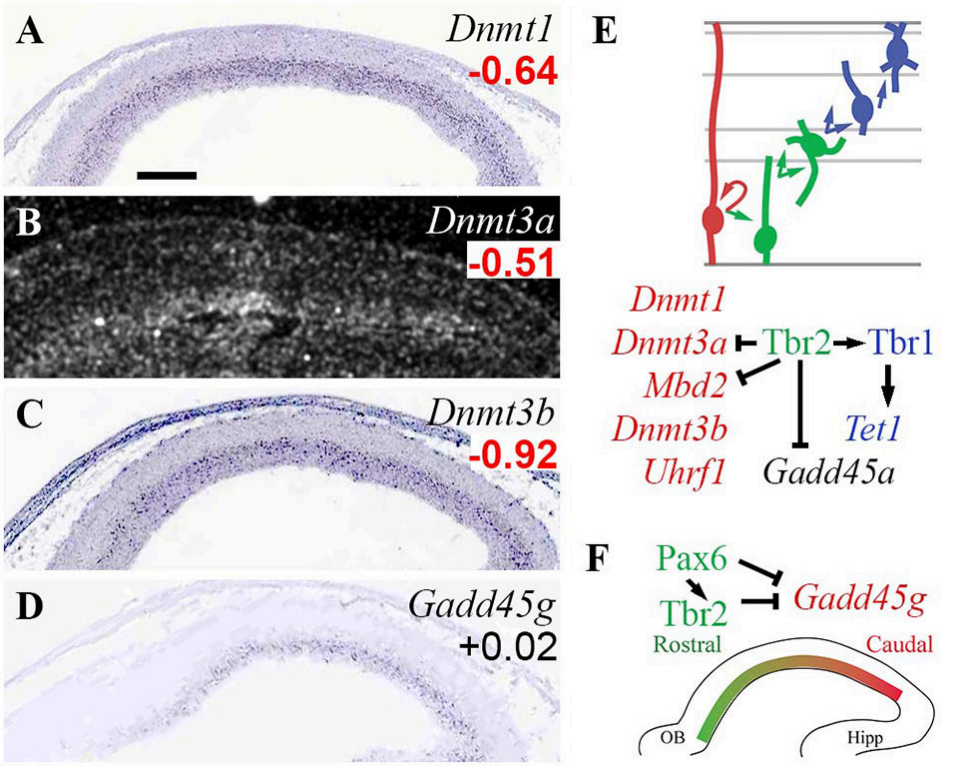
Expression and regulation of DNA methylation/demethylation factors. **(A–D)** Expression of the indicated genes in E14.5 mouse neocortex. *Dnmt1* **(A)**, *Dnmt3a* **(B)**, and *Dnmt3b* **(C)** were expressed in VZ, and were significantly enriched in *Tbr2*-GFP^−^ cells, defining them as RGP markers (Supplementary Table S2). *Gadd45g* **(D)**, part of a pathway for DNA demethylation, was expressed in a high caudal gradient in the VZ, but was not significantly enriched in RGPs or IPs on microarray. (Significant log_2_FC values are indicated by **bold** text, in red or green). Sagittal sections, rostral left, ventral down (see also Supplementary Figure S1). ISH: Genepaint **(A,C,D**) and BGEM (**B**; darkfield). Scale bar: 100 μm. **(E)** Cell-type-specific gene expression and regulation by TFs. Arrows, direct transcriptional activation; bars, direct repression. **(F)** Pax6 and Tbr2 may shape the *Gadd45g* gradient by direct repression.

In the present analysis, all three *Dnmt* genes (*Dnmt1*/*3a*/*3b*) were specifically enriched in RGPs (Figure 2). In addition, *Mbd2* and *Uhrf1* were enriched in Tbr2-GFP^−^ cells, but they were not detected on ISH, and could not be assigned RGP identity with confidence. Downregulation of DNA methylation activity in IPs was directed in part by Tbr2, which directly repressed *Dnmt3a*. Also, *Mbd2* was directly repressed by Tbr2, consistent with the possibility that *Mbd2* is RGP-specific, and actively repressed upon IP differentiation.

Among DNA demethylation genes, *Gadd45g* was regionally enriched with a high caudal gradient in VZ/SVZ, and was directly repressed by Pax6 and Tbr2 (Figure 2F). *Gadd45a*, although not detected by ISH, was also directly repressed by Tbr2 (Figure 2E). *Tet1* was significantly enriched in *Tbr2*-GFP+ cells (although not detected on ISH), and was directly activated by Tbr1.

*Mecp2*, a methyl-cytosine reader linked to Rett syndrome (Qiu, 2017), was enriched in *Tbr2*-GFP+ cells (log_2_FC = +0.72), but not in any specific cell type, as ISH showed high levels in multiple zones. During embryonic neurogenesis, Mecp2 is necessary to limit Pax6 expression in Tbr2+ IPs, and to modulate the pace of PN maturation (Cobolli Gigli et al., 2018).

These results indicate that DNA methylation activity is mainly enriched in RGPs, and that PN differentiation is associated with reduced DNA methylation, and increased DNA demethylation. Also, the high caudal gradient of *Gadd45g* in progenitor zones implicates DNA demethylation in cortical regionalization. Pax6, Tbr2, and Tbr1 regulate this system by repressing and activating key genes, including repression of the caudal marker (*Gadd45g*) by Pax6 and Tbr2 (Figure 2F). Thus, DNA methylation and demethylation may regulate not only neuron differentiation (Sharma et al., 2016) and astrogenesis (Fan et al., 2005), but also cortical regionalization under the control of Pax6 and Tbr2.

### Histone marks

Histone marks are covalent modifications associated with regulation of chromatin structure and transcriptional activity (Allis and Jenuwein, 2016; Gates et al., 2017). Histone marks include acetylation, methylation, ubiquitylation, sumoylation, phosphorylation, and crotonylation. Generally, histone marks are placed by multisubunit enzyme complexes, are recognized by reader proteins, and are reversible by other enzyme complexes. Many EFs that place or remove histone marks have multiple subunit isoforms encoded by different genes, expressed in specific tissues or differentiation stages.

#### Histone acetylation and deacetylation

Histone lysine acetylation generally opens chromatin and activates transcription, while deacetylation represses transcription. Many families of histone acetyltransferases (HATs) and deacetylases (HDACs) mediate placement and reversal of the acetyl marks (Hodawadekar and Marmorstein, 2007; Bannister and Kouzarides, 2011; Sapountzi and Côté, 2011; Drazic et al., 2016). Type-A HATs, such as those in the MYST (e.g., Morf; *Kat6b*), GNAT (e.g., Gcn5; *Kat2a*), and Cbp/p300 (*Crebbp*/*Ep300*) families, regulate transcription and, in some cases, may also acetylate non-histone proteins. Some type-A HATs, such as p300, function as modular activating units that can be recruited by various EF/TF complexes, such as non-canonical PRC1-Auts2 (Gao et al., 2014). Type-B HATs (*Hat1, Hat2*) function in cytoplasmic nucleosome biogenesis. Likewise, some class I HDACs (*Hdac1/2*) serve as modular repressive units, in complexes such as NuRD and Rest/CoRest.

**Figure 3:**
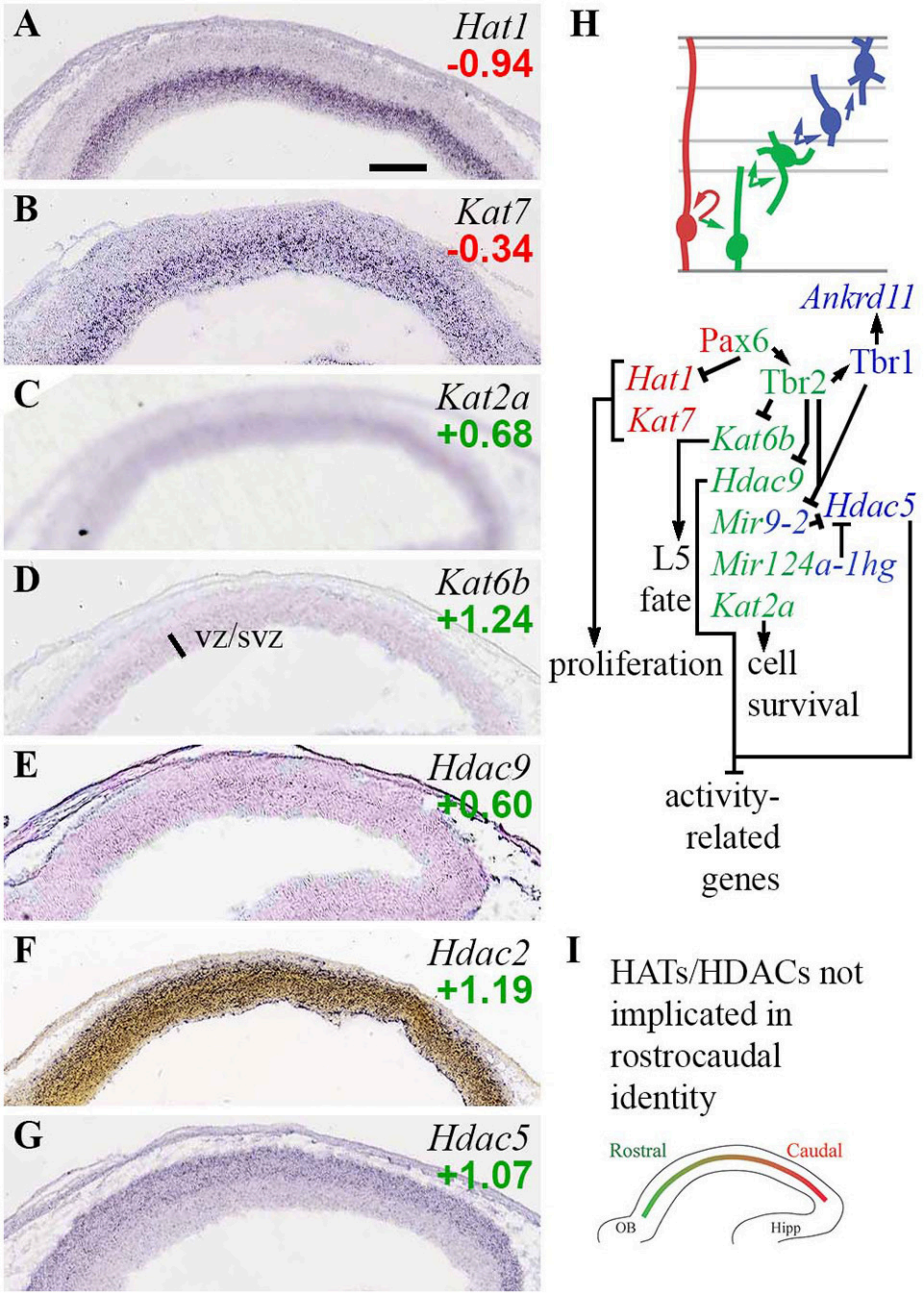
Histone acetylases (HATs) and deacetylases (HDACs). **(A–G)** Expression of indicated genes. *Hat1* **(A)** and *Kat7* **(B)** were RGP-specific. Interestingly, *Hdac9* **(E)** and *Hdac5* **(G)** showed complementary zonal expression in VZ/SVZ and IZ/CP, respectively. ISH: Genepaint **(A–E,G)** and Allen Institute (**F**). Scale bar: 100 μm. **(H)** Cell-type-specific gene expression and regulation. Tbr2, Tbr1, and *Mir9-2* regulate a switch from *Hdac9* in progenitors, to *Hdac5* in PNs. **(I)** No HATs or HDACs exhibited regional expression gradients.

The present analysis identified several HATs and HDACs with cell-type-specific expression, and extensive regulation by Pax6, Tbr2, and Tbr1 (Figure 3). Among HATs, *Hat1* and *Kat7* were RGP-specific. *Hat1* encodes a type-B HAT important in cell proliferation, while *Kat7* (*Myst2*; HBO1), an H3K14 acetyltransferase, is required for general transcriptional activation, especially in progenitor cells during embryonic development (Kueh et al., 2011). *Hat1* was directly repressed by Pax6, and indeed was among the top 100 upregulated genes in *Pax6* null cortex (log_2_FC = +0.84; *p* = 2 × 10^−4^). Type-A HATs Cbp (*Crebbp*) and p300 (*Ep300*) were highly expressed in cortex, but without clear zonal specificity on ISH; nor were they directly regulated by Pax6, Tbr2, or Tbr1.

Two type-A HATs, *Kat2a* and *Kat6b*, were specifically enriched in aIPs and bIPs in the VZ/SVZ (Figures 3C,D). *Kat2a* (Gcn5) is required to prevent apoptosis (Wu et al., 2017). *Kat6b* (*Myst4*; querkopf, Morf), despite being an aIP and bIP marker (Supplementary Table S2), was directly repressed by Tbr2 (Figure 3H; Supplementary Table S4). Interestingly, *Kat6b* is essential for the differentiation of layer 5 neurons (Thomas et al., 2000), and *Tbr2* cKO cortex shows an expanded layer 5 (Mihalas et al., 2016). Also, mice lacking *Brpf1*, an activator of Morf (*Kat6b*), have thin cortex, especially layer 5, and reduced numbers of Tbr2+ IPs (You et al., 2015). Thus, layer 5 differentiation is regulated by a network that includes Tbr2, Morf (*Kat6b*), and Brpf1.

Among HDACs, *Hdac9* (Mitr; an *Hdac* family member without deacetylase activity) was specifically expressed in aIPs and bIPs (Figure 3E; Supplementary Table S2), and was potently repressed by Tbr2 (Supplementary Table S4). In *Tbr2* cKO mice, *Hdac9* was one of the top 100 upregulated genes (log_2_FC = +0.55, *p* = 2 × 10^−4^ on MA1; log_2_FC = +0.68, *p* = 0.008 on MA2). One function of Mitr (*Hdac9*) is to limit gene expression driven by Mef2 and physiological excitation (Méjat et al., 2005). In the context of IPs, we speculate that Mitr might negatively regulate HDAC signaling.

Another HDAC, *Hdac5*, was specifically expressed by PNs in IZ/CP. Recent studies suggest that Hdac5 limits the expression of Mef2c target genes, thus restraining neurite outgrowth (Gu et al., 2018). In turn, *Hdac5* has been identified as a target of miR-124 and miR-9 (Gu et al., 2018), elements of the ncRNA system in developing neocortex (described below). This is noteworthy because both Tbr1 and Tbr2 directly repress *Mir9-2* (host gene of miR-9), and thus indirectly potentiate *Hdac5* expression. *Hdac3* was moderately enriched in *Tbr2*-GFP+ cells, and widely expressed on ISH.

Of the class I HDACs, *Hdac2* was enriched in *Tbr2*-GFP+ cells, and was expressed predominantly by differentiating neurons in the IZ/CP of cortex (Figure 3F), and other forebrain regions (not shown). Thus, *Hdac2* was classified as a marker of general neuronal differentiation starting in the IZ (N-iz; Supplementary Table S2). In contrast, *Hdac1* showed no lineage bias on *Tbr2*-GFP microarray, and was widely expressed with highest levels in the VZ (see the section on Rest/CoRest complexes, below). In sum, *Hdac1* and *Hdac2* showed complementary enrichment in progenitors and neurons, respectively.

Among related factors in histone acetylation, *Uhrf1*, which recruits Dnmt1 and HATs to chromatin during proliferation (Murao et al., 2014), was RGP-specific, as noted (Figure 2E). *Ankrd11*, a scaffolding molecule that potentiates Hdac3 signaling (Gallagher et al., 2015), was significantly enriched in the neuronal lineage, and was activated by Tbr1.

Together, these results reveal an important genetic circuit in IPs that regulates layer 5 differentiation. Also, *Hdac9* and *Hdac5* seem to play similar roles limiting Mef2- and activity-driven gene expression in mature cells, but their expression and regulation in IPs and new PNs suggest they may possibly have distinct functions during neurogenesis. During the IP-PN transition, both Tbr2 and Tbr1 promote the shift from *Hdac9* to *Hdac5* expression. Tbr2 directly represses *Hdac9*, while Tbr2 and Tbr1 indirectly potentiate *Hdac5* expression, by directly repressing *MiR9-2* and thus limiting targeted degradation of *Hdac5* by miR-9 (Figure 3H). These findings support our view that Tbr2 drives the transition from IP to PN, while Tbr1 drives PN differentiation (Mihalas et al., 2016; Mihalas and Hevner, 2017).

#### Trithorax/COMPASS activating complexes

Another important category of histone marks consists of lysine methylation (mono-, di-, and trimethylation) and demethylation. The best-known epigenetic systems using these marks are Trithorax/COMPASS complexes, which place H3K4 trimethyl (H3K4me3) and other marks at active promoters; and PRC2, which places repressive H3K27me3 marks that silence chromatin. The PRC2 system is furthermore connected to PRC1, which places another silencing histone mark—monoubiquitylation of H2A on K119 (H2AK119u1)—and functions synergistically with PRC2. In *Drosophila*, TrxG and Polycomb group (PcG) systems are considered antagonistic; genes marked with both H3K4me3 (activating) and H3K27me3 (repressive) are considered to be in a “bivalent” state, poised for long-term repression or activation. In mammals, the Trithorax and Polycomb systems have become more complex and diverse, with many tissue-specific isoforms and non-canonical subunits. While TrxG genes (as defined by PcG antagonism) also encompass other classes of molecules, such as chromatin remodelers (Schuettengruber et al., 2011; Moccia and Martin, 2018), those other molecules are classified separately for purposes of this article.

Mammalian TrxG H3K4 methyltransferases form complexes known as COMPASS and COMPASS-like, which include core WRAD proteins (*Wdr5, Rbbp5, Ash2l, Dpy30*) and other subunits (Schuettengruber et al., 2011; Piunti and Shilatifard, 2016). Other TrxG proteins are not H3K4 methyltransferases, but have related functions such as H3K36 methylation (Ash1l), chromatin remodeling, modulation of HATs, and general transcriptional regulation (Schuettengruber et al., 2011). Activating marks placed by TrxG complexes can be reversed by demethylation, for example, by Jarid1b (*Kdm5b*) and Lsd1 (*Kdm1a*)—both markers of neuronal differentiation beginning in progenitor zones (Supplementary Table S2).

**Figure 4:**
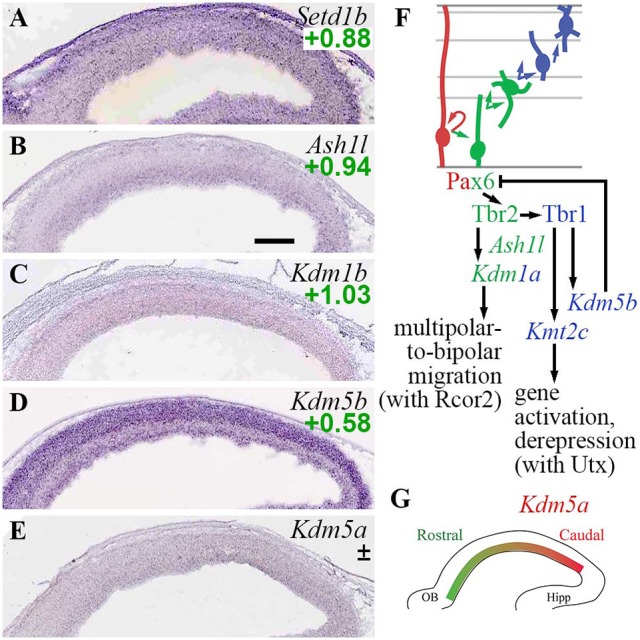
TrxG histone methylation/demethylation EFs. **(A–E)** Expression of indicated genes. The plus/minus symbol (±) indicates that different probes for the same gene, in this example *Kdm5a* **(E)**, showed enrichment in both *Tbr2*-GFP+ and *Tbr2*-GFP– cells on microarray (conflicted). ISH: Genepaint. Scale bar: 100 μm. **(F)** Summary of gene expression and regulation. Interestingly, Tbr1 activated both H3K4 methyltransferase (*Kmt2c*; Mll3) and H3K4 demethylase (*Kdm5b*; Jarid1b) genes. **(G)** Expression of *Kdm5a* (high caudal) was not directly regulated by Pax6, Tbr2, or Tbr1.

In the present analysis, both H3K4 methylase and demethylase genes were expressed predominantly in *Tbr2*-GFP+ cells; none were specifically enriched in RGPs (Figure 4). Among H3K4 methyltransferases, *Setd1b* was enriched in *Tbr2*-GFP+ cells (log_2_FC = +0.88), and was expressed at highest levels in CP (Figure 4A). *Kmt2a* (Mll1) was also enriched in *Tbr2*-GFP+ cells (log_2_FC = +1.36), but was not detected on ISH. Likewise, *Kmt2c* (Mll3) was enriched in *Tbr2*-GFP+ cells (log_2_FC = +1.22), but not detected on ISH. Notably, *Kmt2c* was directly activated by Tbr1 (Supplementary Table S4), suggesting that *Kmt2c* (Mll3) is important for PN differentiation. Indeed, mutations in human *KMT2C* have been linked to intellectual disability (Koemans et al., 2017). Interestingly, Mll3 (*Kmt2c*) forms COMPASS-like complexes with Utx (*Kdm6a*), a demethylase that removes repressive H3K27me3 marks placed by PRC2 (Schuettengruber et al., 2011). By directly activating *Kmt2c* (Mll3) expression, Tbr1 may orchestrate not only the placement of activating H3K4me3 marks by Mll3, but also removal of repressive H3K27me3 marks by Utx.

Among H3K4 demethylases, *Kdm1a* (Lsd1) was enriched in *Tbr2*-GFP+ cells (log_2_FC = +1.14), and was directly activated by Tbr2 (Supplementary Tables S2, S4). Functionally, previous studies have found that Lsd1 interacts with CoRest (*Rcor1/2*), a repressor scaffold protein enriched in aIPs and bIPs (see section Rest and CoRest Complexes, below), to promote a shift from multipolar to bipolar migration (Fuentes et al., 2012). By activating *Kdm1a* (Lsd1) expression, Tbr2 may drive this change of migration mode. *Kdm1b* (Lsd2; an H3K4 demethylase) was similarly enriched in *Tbr2*-GFP+ cells (log_2_FC = +1.03), but its expression was not zone-specific on ISH (Figure 4C). Another H3K4 demethylase, *Kdm5b* (Jarid1b), was enriched in neuronal lineages, and was directly activated by Tbr1 (Figures 4D,F). Thus, Tbr1 drives both deposition and removal of H3K4me3 marks, by activating *Kmt2c* (Mll3) and *Kdm5b* (Jarid1b) respectively, to reconfigure the landscape of active promoters in differentiating PNs.

Functionally, Jarid1b (*Kdm5b*) is necessary to remove inappropriate H3K4me3 marks during development, and thereby deactivate neural progenitor genes such as *Pax6* (Albert et al., 2013). Thus, Tbr1-mediated activation of *Kdm5b* may help block inappropriate *Pax6* expression in neurons (Figure 4F). Indeed, *Pax6* was upregulated in *Tbr1* KO cortex, but not quite significantly (*Pax6* log_2_FC = +1.05, *p* = 0.18 on *Tbr1* KO MA1; log_2_FC = +0.20, *p* = 0.054 on *Tbr1* KO MA2).

*Kdm5a* (Jarid1a), another H3K4me3 demethylase, was expressed in a regional gradient (high caudal) in the VZ/SVZ (Figure 4E). On microarray, different *Kdm5a* probes were enriched in *Tbr2*-GFP+ and GFP^−^ cells (conflicted), so expression of *Kdm5a* could not be specifically assigned to RGPs or IPs.

*Ash1l*, an H3K36 methylase that may activate or repress transcription in different contexts (Schuettengruber et al., 2011; Zhu et al., 2016), was highly enriched in aIPs and bIPs (Figure 4B; Supplementary Table S2), but was not regulated by Pax6, Tbr2, or Tbr1.

These results indicate that deposition and removal of TrxG marks are actively regulated by Tbr2 and Tbr1 during neuronal differentiation (Figure 4F). Also, cortical regionalization may be influenced by Jarid1a (*Kdm5a*), without direct regulation by Pax6, Tbr2, or Tbr1 (Figure 4G).

#### Polycomb repressive complex 2

PcG proteins include components of two distinct complexes, PRC1 and PRC2, which deposit different repressive marks on chromatin (Schuettengruber et al., 2007; Simon and Kingston, 2009; Di Croce and Helin, 2013; Schwartz and Pirrotta, 2013). The marks placed by PRC2 can recruit PRC1, although non-canonical forms of PRC1 also function independently of PRC2 or H3K27me3 (Tavares et al., 2012).

In mammals, a variety of PRC2 complexes with different subunit or isoform composition have been identified (Margueron and Reinberg, 2011). Core PRC2 components include Ezh1 or Ezh2 (methyltransferases), Eed, and Suz12. Canonical PRC2 complexes also contain Rbbp4 or Rbbp7 scaffold proteins. Non-canonical subunits (not found in all PRC2 complexes) can include PCL1-3 proteins (*Phf1, Mtf2, Phf19*, respectively), and Aebp2 or Jarid2. PRC2 also interacts with or is regulated by other EFs, such as Chd4 (Sparmann et al., 2013) and Chd5 (Egan et al., 2013). The repressive H3K27me3 marks placed by PRC2 can be erased by demethylases Utx (*Kdm6a*) and Jmjd3 (*Kdm6b*).

Previously, the PRC2 system has been shown to regulate the timing of neurogenesis in developing neocortex. RGPs lacking Ezh2 undergo accelerated differentiation to produce IPs and neurons, followed by precocious gliogenesis (Pereira et al., 2010). Moreover, *Tbr2* and other key IP-genic or neurogenic genes are marked by high levels of H3K27me3 in RGPs, but these repressive PRC2 marks are removed during IP or neuron differentiation (Albert et al., 2017). PRC2 also regulates rostrocaudal patterning of cortex, as *Suz12* heterozygous null mice have reduced occipital cortex (Miró et al., 2009).

**Figure 5:**
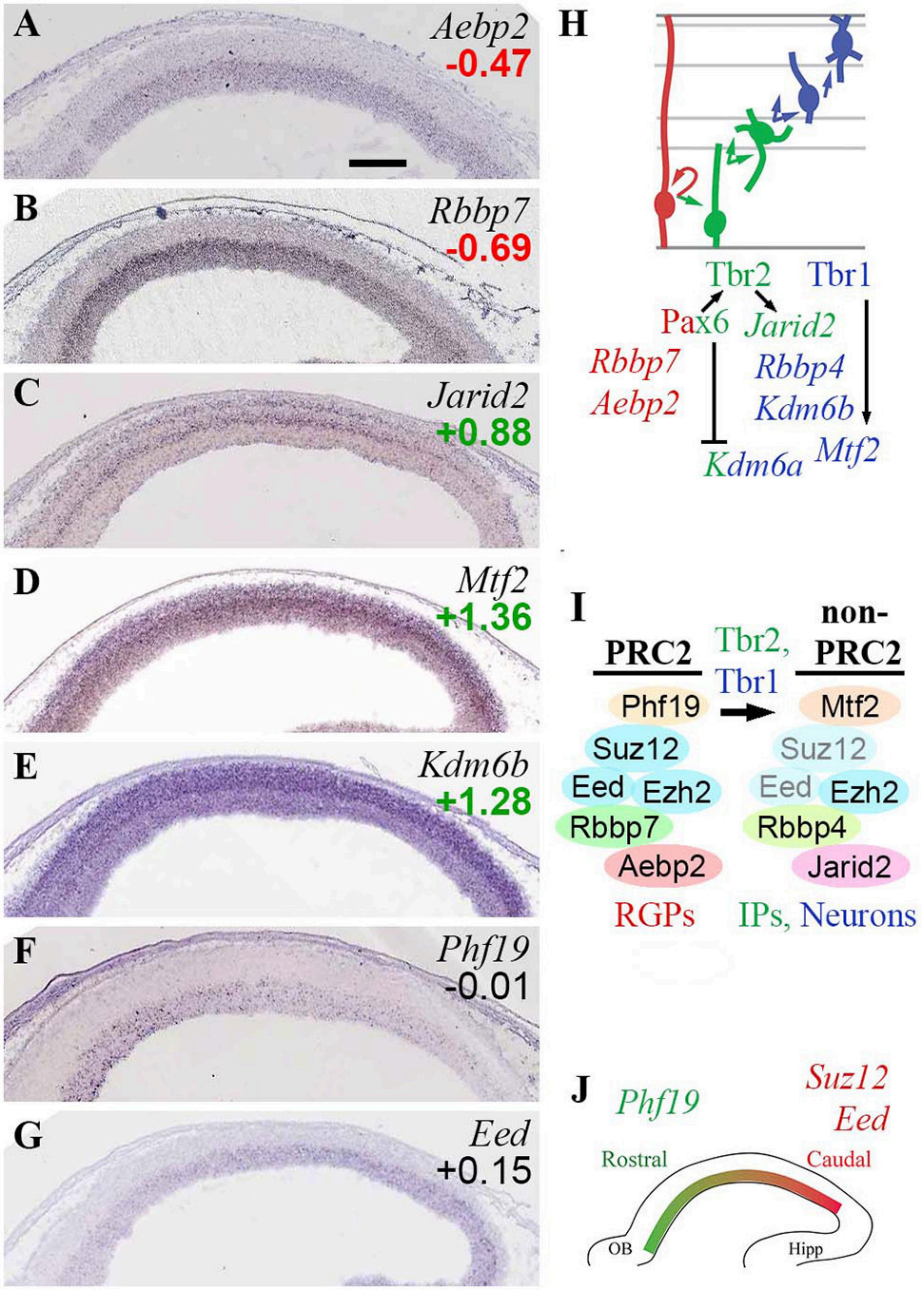
PRC2 complexes. **(A–G)** Expression of indicated genes. ISH: Genepaint. Scale bar: 100 μm. **(H)** Summary of subunit gene expression and regulation. **(I)** Changes in PRC2 subunit expression were associated with PN differentiation, and were regulated by Tbr1/2. Eed and Suz12 subunits were downregulated in differentiating cells (transparent subunits), potentially leading to formation of “non-PRC2” Ezh2 complexes. **(J)** Graded expression of PRC2 subunits is important in cortex regionalization, but these genes are not under direct control of Pax6, Tbr2, or Tbr1.

In the present study (Figure 5), analysis of core PRC2 subunits showed that *Ezh2* was widely expressed in developing neocortex, with slight enrichment in *Tbr2*-GFP+ cells; while *Ezh1* was not detectable. In contrast to the widespread expression of *Ezh2*, the other core PRC2 subunits *Suz12* and *Eed* were expressed almost exclusively in VZ/SVZ, although neither was specifically enriched in *Tbr2*-GFP+ or GFP– cells. Moreover, both *Suz12* (Miró et al., 2009) and *Eed* (Figure 5G) exhibited high caudal to low rostral gradients within VZ/SVZ.

The gradient of *Suz12* expression has previously been linked to cortical regionalization. In *Suz12* heterozygous null mice, occipital cortex was greatly reduced, suggesting that high PRC2 activity instructs occipital identity (Miró et al., 2009). With parallel gradients of core *Suz12* and *Eed* subunit genes, overall PRC2 activity may be steeply graded within the VZ/SVZ. Also, the low levels of *Suz12* and *Eed* expression outside progenitor compartments suggest that PRC2 activity may be essentially limited to the VZ and SVZ.

Other canonical and non-canonical subunits of PRC2 also displayed cell-type-specific or regional expression patterns. *Rbbp7* was specifically expressed in RGPs (Figure 5B), while *Rbbp4* was enriched in *Tbr2*-GFP+ cells. *Aebp2*, encoding a protein that enhances PRC2 activity on PRC1-marked chromatin, was also specifically expressed in RGPs (Figure 5A). In contrast, *Jarid2* (jumonji), a non-canonical PRC2 subunit that may inhibit PRC2 activity (Shen et al., 2009), was specifically enriched in bIPs (Figure 5C), and was directly activated by Tbr2 (Supplementary Table S4). *Mtf2* (PCL2) was highly enriched in the neuronal lineage (Figure 5D), and was directly activated by Tbr1. *Phf19* (PCL3), which targets PRC2 to H3K36me3-marked chromatin, was expressed in a high rostral gradient in VZ/SVZ (counter to *Suz12* and *Eed*). The *Phf19* (PCL3) countergradient suggests that not only the abundance of PRC2 complexes, but also the formation of non-canonical PRC2 complexes, are regionally modulated within VZ/SVZ.

H3K27me3 demethylases Utx (*Kdm6a*; log_2_FC = +0.93) and Jmjd3 (*Kdm6b*; log_2_FC = +1.28) were both enriched in the *Tbr2*-GFP+ PN lineage, but ISH was not available for *Kdm6a*, and *Kdm6b* did not exhibit strict zonal expression (Figure 5E). Importantly, Jmjd3 (*Kdm6b*) interacts with Tbr2 in IPs to derepress neuronal differentiation genes, such as *Tbr1* (Sessa et al., 2017). *Kdm6a* (Utx) was directly repressed by Pax6.

These results suggest that PRC2 complexes undergo extensive subunit switching during differentiation, with overall reduction or loss of canonical PRC2 activity in IPs and neurons (Figures 5H,I). In RGPs, PRC2 likely contains Rbbp7, Aebp2, and PCL3 (*Phf19*) in addition to core subunits. Outside the proliferative zones, *Suz12* and *Eed* are expressed very little, and PCL2 (*Mtf2*) is upregulated in neurons by Tbr1, leaving Ezh2 to potentially form non-PRC2 complexes (Schwartz and Pirrotta, 2013). In IPs, PRC2 activity may be actively suppressed by Tbr2-driven expression of *Jarid2*, an inhibitory subunit (Shen et al., 2009).

Previously, Jarid2 has been associated with Aebp2-containing PRC2 complexes (Schwartz and Pirrotta, 2013; Grijzenhout et al., 2016), but in E14.5 neocortex, *Aebp2* and *Jarid2* showed virtually non-overlapping expression in RGPs and IPs, respectively (Figures 5A,C). Without Aebp2, Jarid2 can nevertheless form alternative PRC2 complexes (Grijzenhout et al., 2016).

Overall, differentiation of IPs and neurons was associated with upregulation of *Kdm6a* (Utx) and *Kdm6b* (Jmjd3), which “unlock” chromatin by remove the H3K27me3 marks placed by PRC2. For regionalization, high canonical PRC2 activity is necessary in caudal VZ/SVZ for occipital cortex identity (Miró et al., 2009), but non-canonical PRC2 is also implicated in regionalization, by the high rostral gradient of *Phf19* (PCL3). Despite the important role of PRC2 in regionalization, the subunits with graded expression are not directly regulated by Pax6, Tbr2, or Tbr1 (Figure 5J).

#### Polycomb repressive complex 1

PRC1 catalyzes monoubiquitylation of H2A lysine 119 (H2AK119u1), and drives chromatin compaction (Schuettengruber et al., 2007; Simon and Kingston, 2009; Di Croce and Helin, 2013; Schwartz and Pirrotta, 2013). Core subunits of canonical PRC1 include: Ring1a (*Ring1*) or Ring1b (*Rnf2*) E3 ligase; PcG ring finger (Pcgf) 2 or Pcgf4 (*Bmi1*); chromobox1-8 (Cbx1-8); Hph1-3 (*Phc1-3*); and substoichiometric amounts of Scm (*Scmh1*/*2*) (Margueron and Reinberg, 2011; Gao et al., 2012; Tavares et al., 2012; Di Croce and Helin, 2013; Schwartz and Pirrotta, 2013). The multiple isoforms of each subunit produce diverse canonical PRC1 complexes.

Non-canonical PRC1 complexes contain Rybp or Yaf2 instead of Cbx, and may contain canonical (Pcgf2/4) or non-canonical (Pcgf1/3/5/6) Pcgf proteins (Gao et al., 2012; Gil and O'Loghlen, 2014; Almeida et al., 2017). In developing cortex, a non-canonical PRC1-Auts2 complex has been described (Gao et al., 2014). Composed of Auts2, Ring1b, Pcgf3/5, Rybp, and casein kinase 2 (CK2), PRC1-Auts2 recruits p300 (*Ep300*), a type-A HAT, to activate (not repress, as usual for PRC1) transcription.

In developing neocortex, PRC1 is thought to regulate the tempo of differentiation, and the balance of neuron subtypes. In Ring1b (*Rnf2*)-deficient RGPs, neurogenesis is prolonged (Hirabayashi et al., 2009), and Ctip2+ layer 5 neurons are increased at the expense of upper layer neurons due to impaired repression of *Fezf2* (Morimoto-Suzki et al., 2014). Non-canonical PRC1-Auts2 complexes are implicated in mouse behavioral development (Gao et al., 2014). In humans, *AUTS2* is an important intellectual disability and autism gene (Beunders et al., 2016).

**Figure 6:**
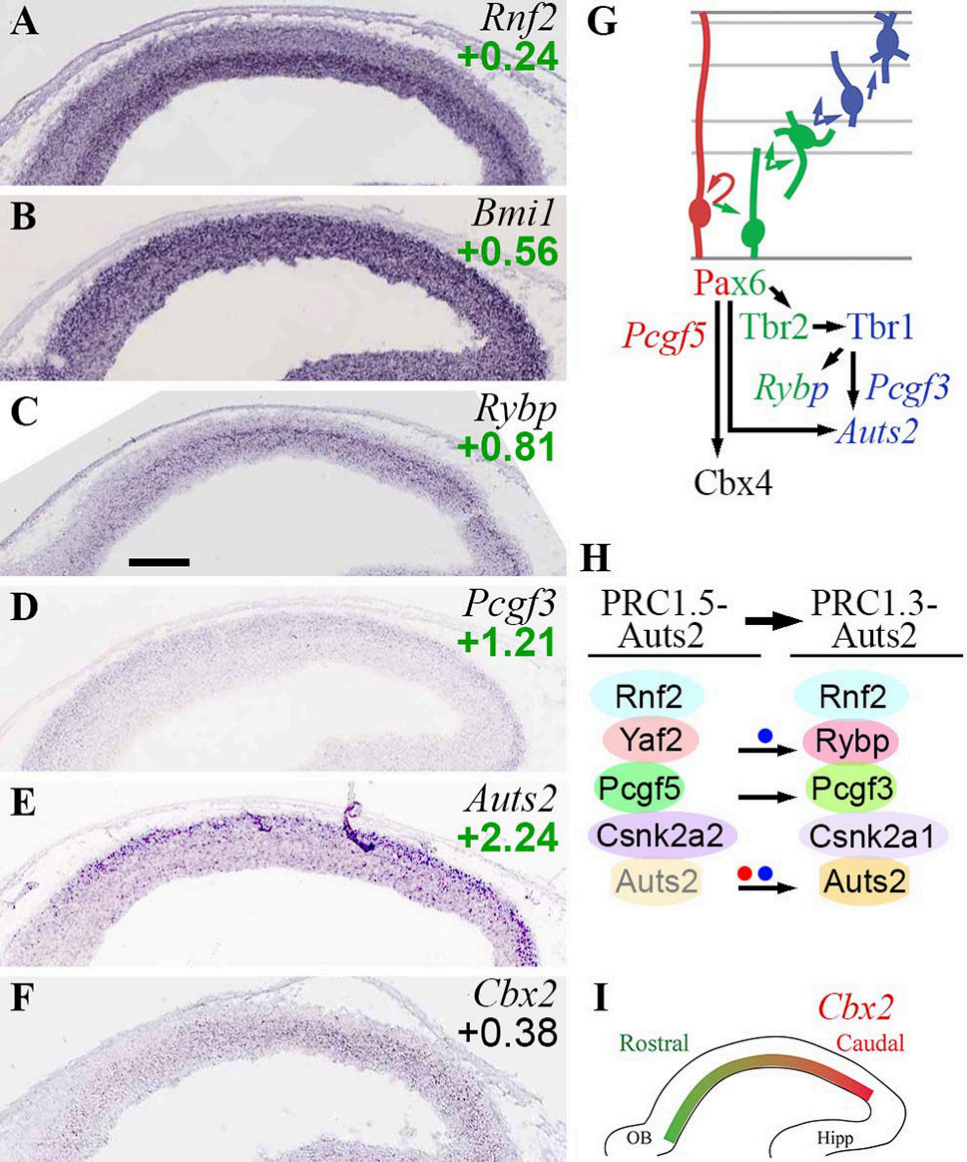
PRC1 complexes. **(A–F)** Expression of indicated genes. ISH: Genepaint. Scale bar: 100 μm. **(G)** Summary of gene expression and regulation. **(H)** Changes in PRC1 subunit expression, and formation of non-canonical PRC1, were associated with PN differentiation, and were regulated by Pax6 (red dot) and Tbr1 (blue dots). *Auts2* was expressed at low levels in VZ/SVZ (transparent Auts2 subunit). **(I)** Graded expression of *Cbx2* (high caudal) was not regulated by Pax6, Tbr2, or Tbr1.

In the present analysis, *Rnf2* (Ring1b) appeared to be the predominant E3 ligase in developing neocortex. *Rnf2* was enriched in *Tbr2*-GFP+ cells, and was seen in all zones by ISH, though highest in the VZ (Figure 6A). In contrast, *Ring1* (Ring1a) was barely detectable on microarrays and ISH.

Canonical PRC1 subunits were, for the most part, widely expressed and little regulated by Pax6, Tbr2, or Tbr1. *Bmi1* (Pcgf4; Figure 6B) and *Pcgf2* were both detected in all zones of neocortex, but highest in VZ. Also, *Bmi1* (Pcgf4) was moderately enriched in *Tbr2*-GFP+ cells, and more highly expressed than *Pcgf2*. Multiple *Cbx* genes were expressed in developing neocortex, but none exhibited cell-type specificity. However, *Cbx4* was directly activated by Pax6. Since Cbx4 promotes sumoylation of Dnmt3a (Li et al., 2007), the upregulation of *Cbx4* by Pax6 may suppress *de novo* DNA methylation during IP genesis. *Cbx2* was expressed in a high caudal gradient in VZ/SVZ (Figures 6F,I). *Phc1-3* were enriched in *Tbr2*-GFP+ cells, but none showed cell-type specificity by ISH. Overall, these findings are consistent with previous studies of PRC1 gene expression in embryonic mouse cortex (Vogel et al., 2006).

Several non-canonical PRC1 subunits exhibited cell-type-specific expression. *Pcgf5* was specifically enriched in RGPs (Supplementary Table S2). In contrast, *Pcgf3* was expressed mainly in the *Tbr2*-GFP+ lineage, especially new neurons (Figure 6D). Similarly, the CK2 alpha isoform switched from alpha-2 (*Csnk2a2*) in progenitors, to alpha-1 in neurons (*Csnk2a1*). *Rybp* was highly enriched in aIPs and bIPs (log_2_FC = +0.81), and was expressed at lower levels in neurons (Figure 6C). *Rybp* was also identified as an IP-specific gene in a previous study (Telley et al., 2016). Significantly, *Rybp* was directly activated by Tbr1 (Supplementary Table S4). *Auts2* was enriched in CP neurons (Figure 6E), but was also expressed at lower levels in VZ/SVZ progenitors (Bedogni et al., 2010b). *Auts2* was directly activated by Tbr1 and Pax6 (Supplementary Table S4; see also Bedogni et al., 2010a).

These data suggest that canonical PRC1 complexes are present in all types of cortical cells (although most abundant in progenitors), and are minimally regulated by Pax6→ Tbr2→ Tbr1. In contrast, non-canonical PRC1 complexes exhibit differentiation-related changes, such as upregulation of *Rybp* in IPs and new PNs. Notably, Tbr1 directly activated two non-canonical PRC1 subunits (*Rybp, Auts2*) implicated in brain development (Gao et al., 2014).

#### Other histone marks and factors

**Figure 7:**
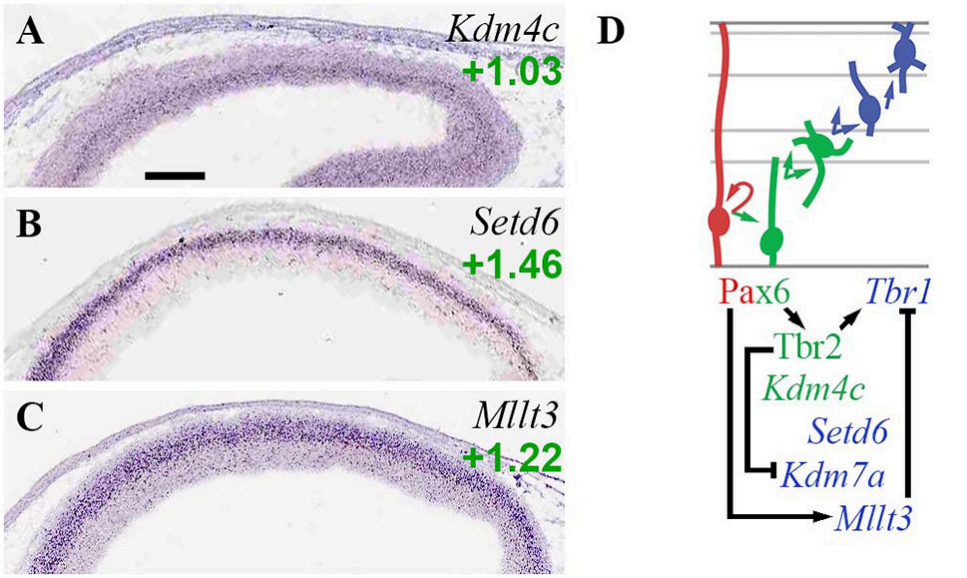
Other histone marks and EFs. **(A–C)** Expression of indicated genes. Interestingly, *Set6d* **(B)** was specifically and exclusively expressed by PNs in developing forebrain. ISH: Genepaint. Scale bar: 100 μm. **(D)** Gene expression and regulation. Notably, Pax6 activated *Mllt3* to indirectly repress *Tbr1*.

*Kdm4c* (Jmjd2c), which encodes an enhancer-associated H3K9 demethylase and scaffold that primes cells for differentiation (Tomaz et al., 2017), was specifically enriched in aIPs and bIPs (Figure 7A; Supplementary Table S2). *Setd6*, an H2AZK7 methyltransferase that confers repressive histone marks, was specifically enriched in migrating PNs in IZ/CP (Figure 7B; see also Supplementary Figure S1F). *Kdm7a* (ISH not available) was enriched in *Tbr2*-GFP+ lineages (log_2_FC = +0.55), but was repressed by Tbr2 (Figure 7D; Supplementary Table S4).

*Mllt3* (Af9), a histone H3K9ac reader, was enriched in neurons of the IZ and CP (Figure 7C). Previously, Af9 has been reported to inhibit deep layer identity by repressing *Tbr1* transcription (Büttner et al., 2010). In the present study, we found that Pax6 directly activated *Mllt3* (Supplementary Table S4). Since previous studies have also found that Pax6 drives upper layer identity (Schuurmans et al., 2004), it seems plausible that Pax6 indirectly represses *Tbr1* by activating high expression of *Mllt3* in precursors of upper layer neurons. Thus, Pax6 indirectly activates *Tbr1* via *Tbr2*, and indirectly represses *Tbr1* via *Mllt3* (Figure 7D).

### ATP-dependent chromatin remodeling complexes

Chromatin remodeling complexes use ATP to modify the positioning, conformation, and isoform composition of histones in nucleosomes—and thereby alter the availability of genes for TF binding (reviewed by López and Wood, 2015; Hota and Bruneau, 2016). These types of complexes contain an Snf2-domain ATPase, along with other proteins that modulate the ATPase activity and confer chromatin target specificity.

In mammals, four main types of chromatin remodeling complexes have been identified: BAF (Brm/Brg1-associated factor), ISWI (Imitation Switch), CHD (chromodomain helicase DNA-binding), and INO80 (inositol auxotrophy 80). The complexes are defined by their ATPase subunits: Brm (*Smarca2*) or Brg1 (*Smarca4*) in BAF (Son and Crabtree, 2014; Sokpor et al., 2017); Snf2h (*Smarca5*) or Snf2l (*Smarca1*) in ACF/CHRAC and NuRF types of ISWI complexes, respectively (Bao and Shen, 2007; Yadon and Tsukiyama, 2011); Chd1-9 alone or in CHD complexes, such as Chd3/4/5 in NuRD (Sims and Wade, 2011; Basta and Rauchman, 2015); and Ino80, Srcap, or p400 (*Ep400*) in INO80 complexes (Gerhold and Gasser, 2014; Hota and Bruneau, 2016).

Most chromatin remodeling complexes contain multiple subunits: up to 16 in BAF, 4 in ISWI, 7 in CHD (NuRD), and 15 in INO80 complexes (Hota and Bruneau, 2016). Some subunit isoforms exhibit tissue-specific or differentiation-related expression. For example, BAF complex subunits are extensively switched in cortical differentiation (Son and Crabtree, 2014).

Besides these large complexes, other ATP-dependent chromatin remodelers, such as Atrx (a Snf2-type ATPase and histone reader protein that places H3.3 in heterochromatin) are also implicated in epigenetic regulation of neurodevelopment (Iwase et al., 2017).

#### ISWI chromatin remodeling complexes

At least eight ISWI complexes have been described in mammals (Goodwin and Picketts, 2017). Furthermore, the ATPase core subunits of ISWI complexes (Snf2h/l) have been shown to be important in brain development. *Smarca1* (Snf2l) mutant mice exhibit excessive, prolonged proliferation of cortical progenitors, especially IPs (Yip et al., 2012); while *Smarca5* (Snf2h) mutant mice exhibit reduced proliferation, at least in cerebellum (Alvarez-Saavedra et al., 2014).

**Figure 8:**
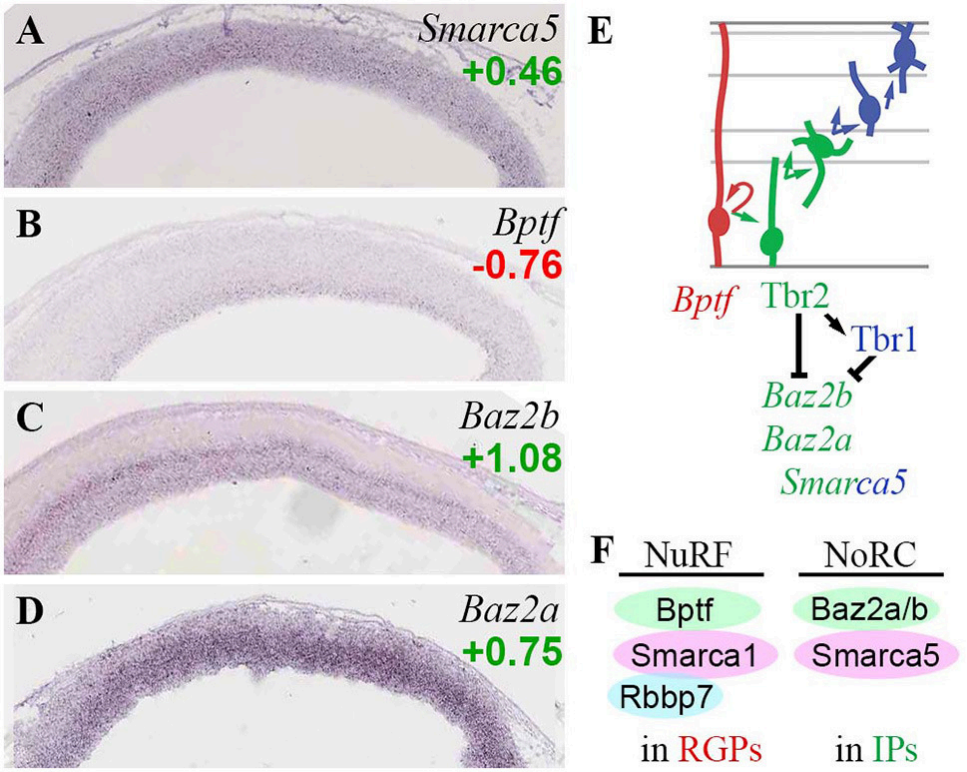
ISWI chromatin remodeling complexes. **(A–D)** Expression of indicated genes. The bilaminar expression of *Baz2b* (**C**) in VZ and SVZ is typical of aIP- and bIP-specific genes (Kawaguchi et al., 2008). ISH: Genepaint. Scale bar: 100 μm. **(E)** Gene expression and regulation. Although *Baz2b* **(C)** is an IP marker, it was directly repressed by Tbr2 and Tbr1 (Supplementary Tables S2, S4). **(F)** NuRF complexes are enriched in RGPs, and NoRC complexes in IPs.

In the present analysis, of the ATPase subunits, *Smarca5* (Snf2h) was expressed in all zones of developing neocortex, with highest levels in VZ/SVZ (Figure 8A), and was overall enriched in neuronal lineages (log_2_FC = +0.46). *Smarca1* (Snf2l) was expressed in multiple zones, and did not show differential expression on *Tbr2*-GFP microarray. Thus, both ISWI ATPases were widely expressed in developing neocortex, although *Smarca5* (Snf2h) was somewhat higher in progenitors. This interpretation matches a previous description (Lazzaro and Picketts, 2001).

*Bptf*, an essential core subunit of NuRF (nucleosome-remodeling factor) complexes, was specifically enriched in RGPs (Figure 8B). In addition to Bptf, NuRF contains not only Snf2l (*Smarca1*), but also either RbAP48 (*Rbbp4*) or RbAP46 (*Rbbp7*) (Qiu et al., 2015). Like *Bptf*, *Rbbp7* was specifically expressed in RGPs (Figure 5B). In contrast, *Rbbp4* was highly enriched in *Tbr2*-GFP+ lineages (log_2_FC = +1.58). These data suggest that NuRF complexes are restricted to RGPs, and are comprised of Bptf/Snf2l/RbAP46 (Figure 8F). Bptf also interacts with Myc to promote cell cycle progression (Richart et al., 2016).

*Baz2b*, a reader that binds H3K14ac as part of an unknown ISWI complex (Bortoluzzi et al., 2017), was specifically expressed in aIPs and bIPs, and was directly repressed by Tbr2 and Tbr1 (Figures 8C,E). *Baz2a* (Tip5), a component of NoRC (nucleolar remodeling complex) in the Snf2h-containing ACF/CHRAC group of ISWI remodelers, was also highly enriched in IPs (Figure 8D). Similarly, *Baz1b* (Wstf) was expressed at high levels in VZ, and was moderately enriched in the *Tbr2*-GFP+ lineage (log_2_FC = +0.85); thus, WICH complexes (Wstf/Snf2h) may be enriched in progenitors, especially IPs.

Overall, the present analysis suggests that NuRF complexes are specifically present in RGPs, while NoRC complexes are particularly abundant in IPs (Figure 8F). The direct repression of *Baz2b* by Tbr2 and Tbr1 suggests that downregulation of some ISWI complexes (possibly a Baz2b-containing NoRC variant) is important for differentiation from IPs to PNs.

#### INO80 chromatin remodeling complexes

Among ATPase subunit genes, *Ino80* was detected primarily in VZ, but was not enriched in *Tbr2*-GFP^−^ or GFP+ lineages (Supplementary Table S3). *Ino80b* (Ies2), which activates the ATPase activity of Ino80, was specifically expressed in RGPs (log_2_FC = −0.45), suggesting that Ino80-containing complexes are enriched and activated in RGPs. The INO80 remodelers are important in DNA replication and repair, as well as transcriptional regulation (Poli et al., 2017), so the enrichment of Ino80 activity in RGPs may be related to high proliferative activity in this cell type.

*Srcap* and *Ep400* (p400) were detected in multiple zones, and were moderately enriched in *Tbr2*-GFP+ cells (log_2_FC = +0.28 for *Srcap*; +0.76 for *Ep400*). Most Srcap complex subunits were widely expressed, while several p400 complex subunits, such as *Kat5* (Tip60), were relatively enriched in neurons. Pax6, Tbr2, and Tbr1 were not implicated in the regulation of INO80 complex subunits.

Together, these findings suggest that Ino80-containing complexes are specifically active in RGPs, while p400/Tip60 complexes are most active in postmigratory CP neurons. The functions of INO80 complexes in cortical development are unknown.

#### CHD chromatin remodeling complexes

**Figure 9:**
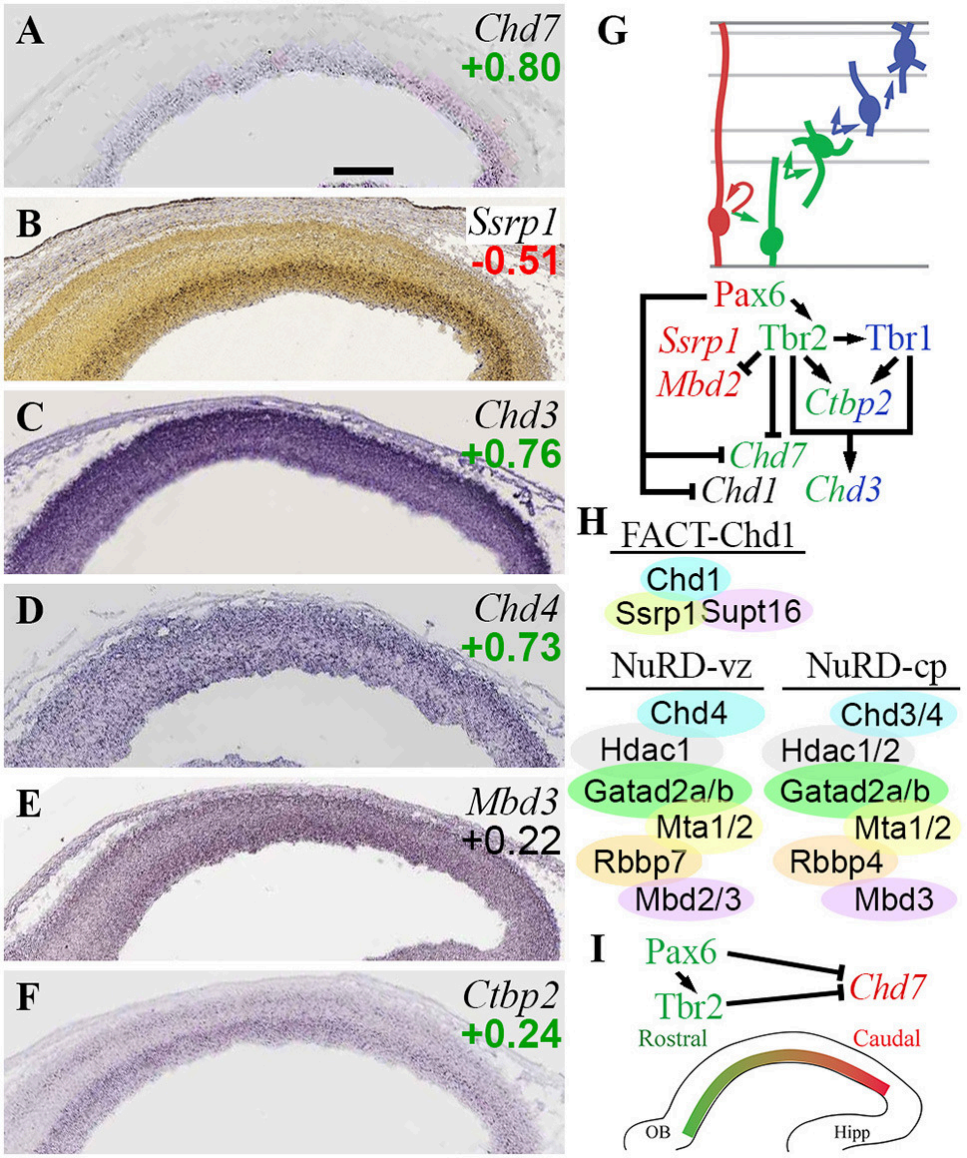
CHD chromatin remodeling complexes. **(A–F)** Expression of indicated genes. ISH: Genepaint **(A,C–F)** and Allen Brain Atlas Developing Mouse Brain **(B)**. Scale bar: 100 μm. **(G)** Gene expression and regulation. Notably, Tbr1 and Tbr2 synergistically activate *Chd3*, a NuRD subunit. **(H)** CHD complexes in E14.5 neocortex include FACT-Chd1 in RGPs, and NuRD in progenitors (Chd4-containing) and neurons (Chd3/4-containing). **(I)** Gradient of *Chd7* expression (high caudal) is shaped by Pax6 and Tbr2.

Among *Chd* ATPase genes, only *Chd7* exhibited cell-type or region-specific expression—indeed, both. *Chd7* was enriched in *Tbr2*-GFP+ cells (log_2_FC = +0.80) on microarray, and was expressed specifically in VZ on ISH, identifying *Chd7* as a specific marker of aIPs. Within VZ, *Chd7* exhibited high caudal expression (Figure 9A), suggesting its involvement in regionalization. Consistent with this possibility, we also found that *Chd7* was directly bound and repressed by Pax6 and Tbr2 (Figure 9I; Supplementary Table S4), both of which promote rostral identity. Previous studies suggest that *Chd7* binds mainly to enhancers and active transcription start sites, and is essential for activation of neuronal differentiation genes (Moccia and Martin, 2018). Mutations in human *CHD7* cause CHARGE syndrome, a complex disorder with significant brain and somatic anomalies (Feng et al., 2017; Moccia and Martin, 2018).

Other *Chd* genes regulated by TFs included *Chd1*, repressed by Pax6; and *Chd3*, jointly activated by Tbr1 and Tbr2. *Chd1* was not specifically enriched in *Tbr2*-GFP+ or GFP– lineages, nor was ISH available, so the topography of *Chd1* expression is unknown. Chd1 protein recognizes H3K4me3 marks (active promoters) and globally activates transcription (Guzman-Ayala et al., 2015). Also, Chd1 interacts with FACT complex (Ssrp1 and Supt16) at centromeres to facilitate histone exchange (Okada et al., 2009). Of the FACT subunits, *Ssrp1* was RGP-specific (Figure 9B), while *Supt16* was widely expressed. These data suggest that FACT-Chd1 complexes may be abundant in RGPs, but downregulated in IPs, in part by Pax6 repression of *Chd1* (Figures 9G,H).

*Chd3* (Figure 9C), directly activated by Tbr1 and Tbr2, encodes a core component of NuRD (nucleosome remodeling deacetylase) complexes. Other core Chd subunits in NuRD include *Chd4* (Figure 9D) or *Chd5* (mutually exclusive alternatives). NuRD complexes have at least six subunits, each of which has multiple alternatives or isoforms: Chd3-5, Hdac1/2; Mbd2/3; Mta1-3; Gatad2a/b; and Rbbp4/7 (Basta and Rauchman, 2015). Recent studies suggest that NuRD complexes are comprised of different Chd proteins during different stages of differentiation (Nitarska et al., 2016). In RGPs, NuRD was found to contain Chd4, Mta2, and Hdac2; in neurons, Chd4 was replaced by Chd3 and Chd5 (Nitarska et al., 2016). Also, NuRD was recently found to interact with Lhx2 to repress layer 5 genes (Muralidharan et al., 2017). Functionally, loss of NuRD components *Mbd3* (Knock et al., 2015) or *Chd4* (Nitarska et al., 2016) cause similar defects of RGP proliferation, leading to reduced IP genesis and thinner cortex. Such phenotypes are consistent with the general function of NuRD complexes in cell cycle progression (Basta and Rauchman, 2015), but much remains to be learned about the control of PN differentiation by NuRD.

Direct activation of *Chd3* by Tbr2 and Tbr1 supports the conclusion that *Chd3* expression increases with neuronal differentiation. In the present analysis, *Chd4* was not, however, specifically enriched in RGPs as previously suggested (Nitarska et al., 2016). Rather, *Chd4* exhibited widespread expression in cortical zones, and *Chd4* was (like *Chd3*) enriched in *Tbr2*-GFP+ cells on microarray (Figures 9C,D), while *Chd5* was essentially undetectable. These data suggest that in RGPs, NuRD complexes contain mainly Chd4, while in neurons, NuRD complexes contain both Chd3 and Chd4 (Figure 9H).

Most other NuRD subunits did not exhibit cell-type-specific expression, but a few did. As noted above, *Mbd2* was specifically enriched in *Tbr2*-GFP^−^ cells (likely RGPs; ISH not informative), and was directly repressed by Tbr2 (Figure 2E; Supplementary Tables S3, S4). In contrast, *Mbd3* was widely expressed (Figure 9E). *Rbbp7* was specifically expressed in RGPs (Figure 5B), while *Rbbp4* was primarily enriched in neuron lineages (see also sections on *Rbbp4/7* in PRC2 and NuRF complexes). *Hdac1* was expressed in all zones but enriched in VZ/SVZ, while *Hdac2* was moderately enriched in neurons (Figure 3F). *Mta1/2* were widely expressed, while *Mta3* was essentially undetectable. *Gatad2a/b* were both enriched in *Tbr2*-GFP+ cells, and *Gatad2a* was widely expressed on ISH, but *Gatad2b* ISH was not available. *Ctbp2*, a NuRD partner that targets it to active genes that require silencing during differentiation (Kim et al., 2015), was directly activated by Tbr2 and Tbr1 (Figures 9F,G).

Overall, these findings suggest that NuRD subunit composition and silencing activity are modulated during differentiation from RGPs to neurons. These changes are driven in part by Tbr2 and Tbr1 (Figures 9G,H). Also, the graded expression of *Chd7*, and its repression by Pax6 and Tbr2, implicate *Chd7* in cortical regionalization (Figure 9I), although further studies will be necessary to substantiate this role.

#### BAF chromatin remodeling complexes

Among EFs with documented importance in cortical development, the BAF chromatin remodeling complex plays a well-established role in regulating cerebral cortex size and function (Narayanan et al., 2015; Sokpor et al., 2017). Moreover, BAF subunit switching occurs at specific stages of neuronal differentiation (Son and Crabtree, 2014). The BAF complex is important for human brain development, as genetic defects of BAF subunits, such as Baf250b (*Arid1b*), cause Coffin-Siris syndrome, a microcephaly disorder with intellectual disability (Son and Crabtree, 2014).

Conserved subunits of BAF between yeast and mice include a core ATPase, consisting of either Brm (*Smarca2*) or Brg1 (*Smarca4*); Baf155/170 (*Smarcc1/2*); Baf60a-c (*Smarcd1-3*); Baf53a/b (*Actl6a/b*); and Baf47 (*Smarcb1*). In addition, mammalian BAF complexes contain ≥10 other subunits, such as Baf250a/b (*Arid1a/b*) (Son and Crabtree, 2014; Hota and Bruneau, 2016).

In cortical development, BAF has been shown to exchange four subunits during differentiation from progenitors to neurons (Son and Crabtree, 2014). Neural progenitor BAF (npBAF) contains Baf53a (*Actl6a*), Ss18, and Baf45a/d (*Phf10*/*Dpf2*); in neuronal BAF (nBAF), these subunits are replaced with Baf53b (*Actl6b*), Crest (*Ss18l1*), and Baf45b/c (*Dpf1/3*), respectively. Interestingly, the shift from Baf53a (*Actl6a*) to Baf53b (*Actl6b*) is driven by microRNA (miR)-9^*^ and miR-124, which target *Actl6a* (Baf53a) for degradation (Son and Crabtree, 2014). In the section on ncRNA, we show that *Mir9-2* (encoding miR-9^*^) is directly repressed by Tbr2 and Tbr1.

**Figure 10:**
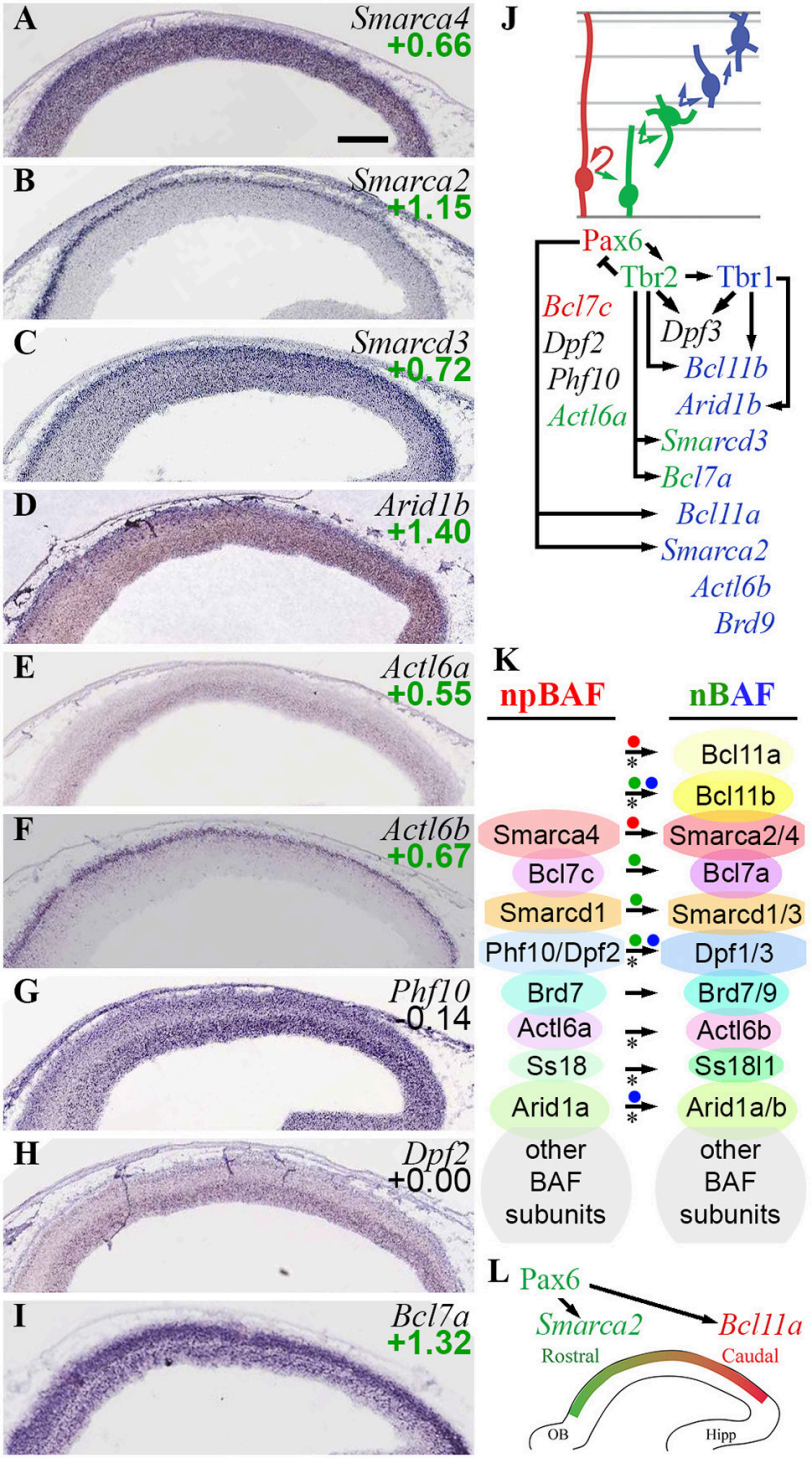
BAF chromatin remodeling complexes. **(A–I)** Expression of indicated genes. Remarkably, *Smarca2* (Brm; **B**) was specifically expressed by postmitotic PNs in the CP, with a high rostral gradient. ISH: Genepaint. Scale bar: 100 μm. **(J)** Summary of gene expression and regulation. **(K)** BAF subunit switching was controlled by Pax6 (red dots), Tbr2 (green dots), and Tbr1 (blue dots). Asterisks: previously described switches in BAF subunit composition, confirmed here. **(L)** *Smarca2* (high rostral in CP) and *Bcl11a* (high caudal in IZ/CP) were both directly activated by Pax6, reflecting multiple functions of Pax6 in cortical development (see text for details).

The present analysis confirmed previously described BAF subunit switching, and found multiple additional subunits that switch during differentiation (Figure 10). Of the core ATPase subunits, *Smarca4* (Brg1) was ubiquitous, but *Smarca2* (Brm) was specifically expressed by postmigratory PNs (Figures 10A,B). *Smarca2* also displayed a high rostral gradient, and was directly activated by Pax6. Among the other core subunits, *Smarcd1* (Baf60a) was ubiquitously expressed, while *Smarcd3* (Baf60c) was enriched in the CP (Figure 10C), and was directly activated by Tbr2 (Figure 10J). Similarly, *Smarcc1* (Baf155) was ubiquitously expressed, while *Smarcc2* (Baf170) was abundant in CP (Supplementary Table S3). Previously, Baf170 (*Smarcc2*) has been linked to repression of IP genesis and neurogenesis (Tuoc et al., 2013).

The previously described (Son and Crabtree, 2014) shift from *Actl6a* (Baf53a) to *Actl6b* (Baf53b) was confirmed on ISH (Figures 10E,F), although enrichment of *Actl6a* in *Tbr2*-GFP+ cells (log_2_FC = +0.55) suggested that *Actl6a* was expressed in not only RGPs, but also IPs. Likewise, npBAF subunits *Phf10* (Baf45a) and *Dpf2* (Baf45d) were highly expressed in VZ/SVZ (Figures 10G,H), while nBAF subunits *Dpf1/3* (Baf45b/c) were highly expressed in IZ/CP. Upregulation of *Dpf3* (Baf45c) in differentiating neurons was directly activated by Tbr1 and Tbr2.

Among the newly observed subunit exchanges, *Bcl7c* (Baf40c) was specifically expressed in RGPs (log_2_FC = −1.07), while *Bcl7a* (Baf40a) was enriched in IPs and neurons (log_2_FC = +1.32). These findings define Baf40c and Baf40a as components of npBAF and nBAF, respectively (Figure 10I; Supplementary Table S2). Moreover, *Bcl7a* expression was directly activated by Tbr2.

Mammalian BAF complexes are sometimes categorized by Baf250 isoform, as Baf250a- (BAF-A) and BAF250b-containing (BAF-B) complexes (Hota and Bruneau, 2016). We observed that *Arid1a* (Baf250a) was ubiquitously expressed, while *Arid1b* (Baf250b) was enriched in the CP (Figure 10D), and was directly activated by Tbr1 (Supplementary Table S4). These results suggest that BAF-A predominates in progenitors, while cortical PNs express BAF-A and BAF-B complexes, the latter driven by Tbr1-mediated activation of *Arid1b*.

A special type of BAF complex, called Polybromo-associated BAF (PBAF), is formed by the incorporation of four specific subunits in Brg1 (*Smarca4*)-containing BAF: Baf180 (*Pbrm1*), Baf200 (*Arid2*), Baf45a (*Phf10*), and Brd7 (St. Pierre and Kadoch, 2017). These genes were generally enriched in progenitor zones (VZ/SVZ) relative to IZ/CP, and were moderately enriched in *Tbr2*-GFP+ cells (Supplementary Table S3). Thus, PBAF may be most abundant in progenitor cells, and decline with PN differentiation. The upregulation of *Smarca2* (Brm) in PNs (Figure 10B) may further diminish the overall formation of PBAF complexes.

Ctip1/Baf100a (*Bcl11a*) and Ctip2/Baf100b (*Bcl11b*) are BAF subunit TFs with major roles in PN differentiation and regionalization (Arlotta et al., 2005; Wiegreffe et al., 2015; Greig et al., 2016; Woodworth et al., 2016). Both *Bcl11a* (log_2_FC = +1.50) and *Bcl11b* (log_2_FC = +1.75) were highly enriched in the *Tbr2*-GFP+ lineage, and both were expressed predominantly in neuronal differentiation zones. Additionally, *Bcl11a* was expressed in a high caudal gradient, as described (Greig et al., 2016). We found that Pax6 directly activated expression of *Bcl11a*, while Tbr2 and Tbr1 directly activated *Bcl11b* (Figures 10J,K). The activation of *Bcl11a* by Pax6 suggests that Pax6 drives *Bcl11a* as part of the programs for neuron migration (Wiegreffe et al., 2015) and subtype specification (Woodworth et al., 2016); the high-caudal *Bcl11a* gradient runs counter to *Pax6* and is presumably shaped by other TFs.

The present results indicate that the subunit composition of BAF complexes is highly regulated in cortical PN differentiation; and that the Pax6→ Tbr2→ Tbr1 cascade is responsible for activation of many BAF subunit genes in IPs and neurons, as well as the activation of *Smarca2* in a high rostral gradient (Figures 10J–L). Interestingly, Pax6, Tbr2, and Tbr1 did not directly repress any npBAF subunit genes. Recently, BAF complexes were reported to interact with Utx (*Kdm6a*) and Jmjd3 (*Kdm6b*), and potentiate their H3K27me3 demethylase activity (Narayanan et al., 2015). Thus, the Pax6→ Tbr2→ Tbr1 cascade drives the formation of two complexes that recruit H3K27me3 demethylases: BAF (Narayanan et al., 2015) and Mll3/COMPASS-like (Schuettengruber et al., 2011).

### Rest and CoRest complexes

A longstanding paradigm of TF-EF interactions is the recruitment of Hdac1/2 by Rest (repressor element-1 silencing TF) to prevent neuronal differentiation (Qureshi et al., 2010). Seminal research showed that Rest binds specific DNA sequences, and recruits corepressor scaffold proteins (CoRest, Sin3) that also bind class I HDACs (*Hdac1/2*), to silence neuronal genes (Ballas et al., 2001; Lunyak et al., 2002). Complicating the picture, two isoforms of CoRest (*Rcor1/2*) have been distinguished, and other CoRest interactions and functions have been discovered (Ooi and Wood, 2007; Qureshi et al., 2010). In developing neocortex, *Rcor1/2* have been implicated in neuron subtype specification (Abrajano et al., 2009) and migration (Fuentes et al., 2012). Some functions of CoRest appear to be mediated by novel complexes with Lsd1 (*Kdm1a*; Fuentes et al., 2012) and Insm1 (Monaghan et al., 2017). The Rcor/Insm1 complex promotes neuronal differentiation, and immature progenitors accumulate in the absence of *Rcor1/2* (Monaghan et al., 2017).

**Figure 11:**
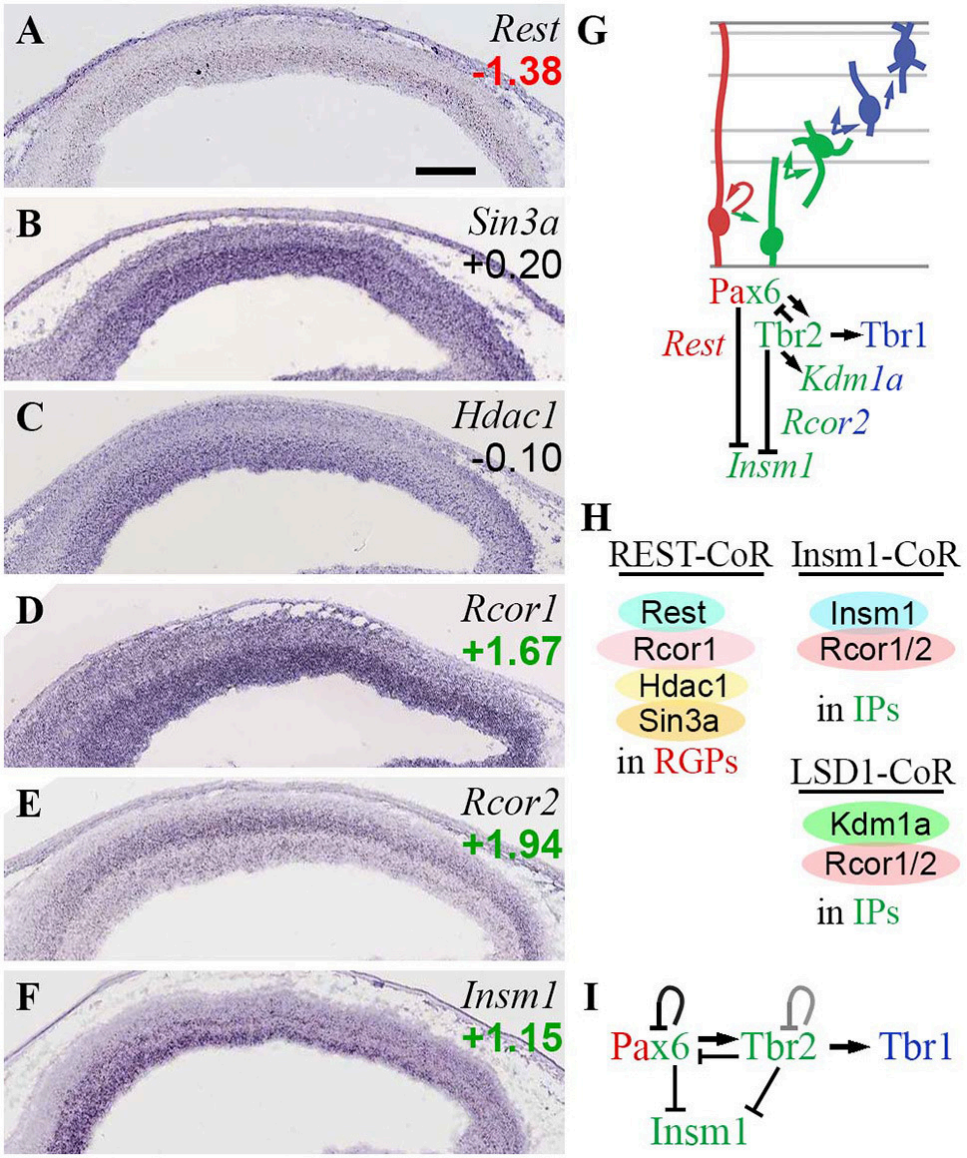
Rest and CoRest complexes. **(A–F)** Expression of indicated genes. While *Rest* **(A)** was specifically expressed in RGPs, CoRest genes *Rcor1* **(D)** and *Rcor2* **(E)** were enriched in IPs, as was *Insm1* **(F)**. ISH: Genepaint. Scale bar: 100 μm. **(G)** Summary of gene expression and regulation. Interestingly, Tbr2 activated *Kdm1a* (Lsd1) but repressed *Insm1*; both are CoRest (*Rcor1/2*) binding partners. Pax6 also repressed *Insm1*. **(H)** Rest/CoRest complexes form in RGPs, while Insm1/CoRest and Lsd1/CoRest complexes form primarily in IPs. **(I)** Repression of *Insm1* by the Pax6→ Tbr2→ Tbr1 cascade. Like Pax6 and Tbr2, Insm1 is a key regulator of IPs (Farkas et al., 2008). The *Tbr2* loop is shown in gray to reflect unknown effect of Tbr2 on its own transcription.

In the present analysis (Figure 11), *Rest* was specifically expressed in RGPs (Figure 11A), consistent with its established function of suppressing neuronal differentiation. Of corepressors, *Sin3a* and *Rcor1* were expressed mainly in VZ (and *Rcor1* was enriched in *Tbr2*-GFP+ cells), while *Rcor2* was expressed mainly in SVZ/IZ and inner VZ (Figures 11B,D,E). The enrichment of *Rcor2* in *Tbr2*-GFP+ cells (log_2_FC = +1.94), together with its bilaminar expression pattern in VZ and SVZ (Figure 11E), indicated specific enrichment in aIPs and bIPs (Supplementary Table S2). Of the interacting HDACs, *Hdac1* was expressed at highest levels in the VZ (Figure 11C), while *Hdac2* was expressed mainly in IZ/CP, and was enriched in *Tbr2*-GFP+ cells (Figure 3F). Thus, Rest/CoRest complexes form predominantly in RGPs, where Rest recruits mainly Sin3a and Hdac1, and possibly Rcor1 (Figure 11H). Interestingly, one function of Rest is to repress miR-9^*^ and miR-124 (Yoo et al., 2009); as shown below in the section on ncRNA, miR-9^*^ is also repressed by Tbr1 and Tbr2.

Of other proposed Rcor1/2-interacting factors, *Kdm1a* (Lsd1) was ubiquitously expressed (Fuentes et al., 2012) and was enriched in *Tbr2*-GFP+ cells (log_2_FC = +1.14). Also, *Kdm1a* (Lsd1) was directly bound and activated byTbr2 (Figure 11G). *Insm1* was expressed mainly in VZ and SVZ (Figure 11F), and was also highly enriched in *Tbr2*-GFP+ cells (log_2_FC = +1.16). In contrast to *Kdm1a* (Lsd1), which was activated by Tbr2, *Insm1* was repressed by both Tbr2 and Pax6 (Figure 11G; Supplementary Table S4). These results suggest that Pax6 and Tbr2 promote the formation of Rcor/Lsd1 complexes regulating PN migration, but suppress IP-genic Rcor/Insm1 complexes (Figures 11G,H).

Importantly, *Insm1* has previously been implicated in the genesis of IPs: *Insm1* null mice have decreased IP abundance, and reduced *Tbr2* expression (Farkas et al., 2008). One function of Insm1 is to promote the delamination of cortical progenitors, by directly repressing *Plekha7* (Tavano et al., 2018). Since Insm1 is thought to be a transcriptional repressor, and directly represses *Rest* (Monaghan et al., 2017), it seems unlikely that Insm1 directly activates *Tbr2*. Nevertheless, Insm1 is an integral component of the TF network regulated by Pax6→ Tbr2→ Tbr1 (Figure 11I).

### Non-coding RNA-mediated epigenetic regulation

Many ncRNA species regulate the expression of target genes at transcriptional or post-transcriptional levels. One well-known example of the former is *Xist*, a long (>200 nt) ncRNA (lncRNA) that binds chromatin to mediate X-inactivation (Almeida et al., 2017). Typically, microRNAs (miRs) target specific mRNAs for degradation (Hsieh and Zhao, 2016; Yao et al., 2016).

**Figure 12:**
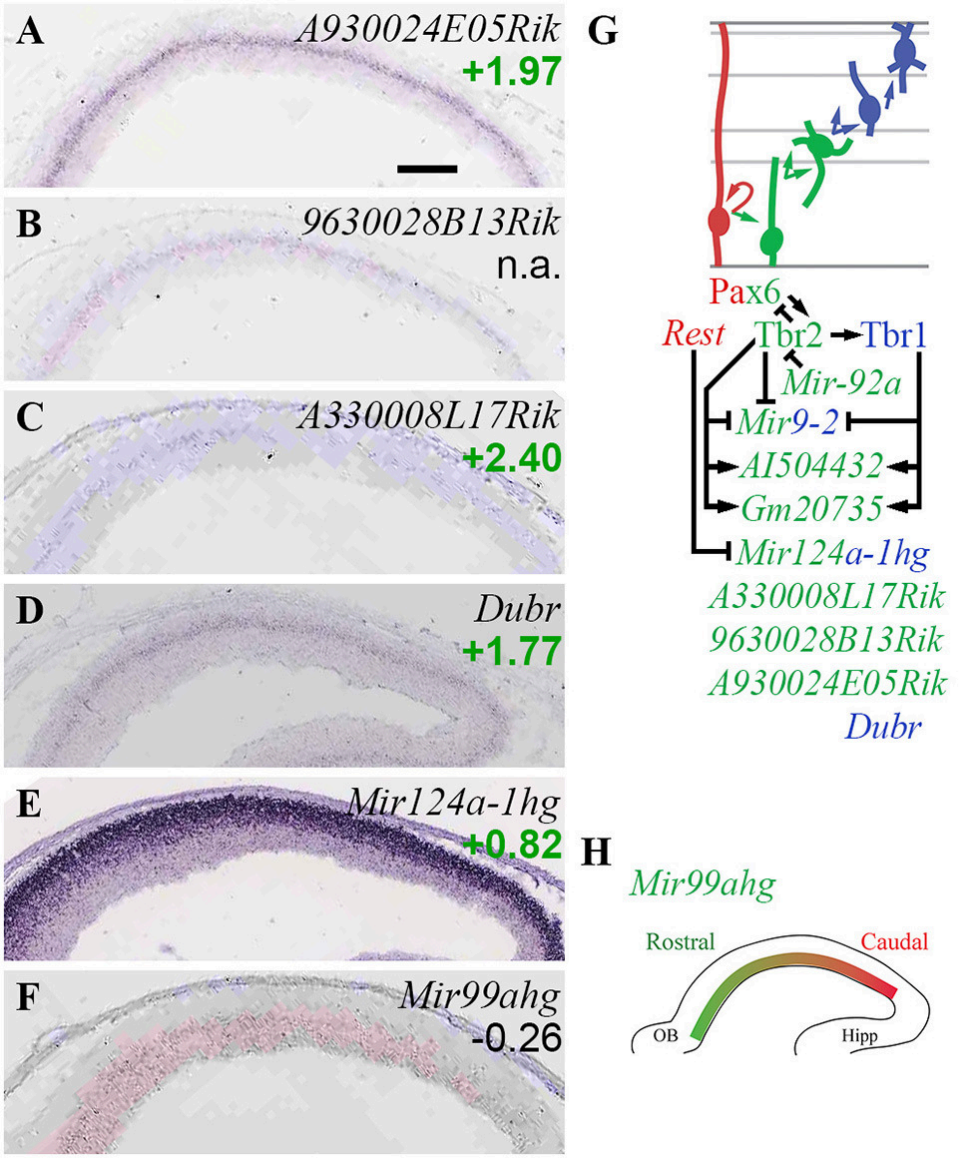
Non-coding RNA. **(A–F)** Expression of indicated genes. Remarkably, several lncRNA genes **(A–D)** showed similar expression patterns in bIPs and/or new neurons in SVZ/IZ. ISH: Genepaint. Scale bar: 100 μm. **(G)** Summary of ncRNA gene expression and regulation. **(H)** Expression of *Mir99ahg* (high rostral) was not regulated by Pax6, Tbr2, or Tbr1; but may shape the high caudal gradient of its target, *Fgfr3* (not shown). See text for details.

Previous studies of developing neocortex have shown that miRs in the miR-17-92 cluster prevent the transition from RGPs to IPs, in part by targeting *Tbr2* and *Cdkn1a* (p21) (Bian et al., 2013; Chen et al., 2014). Within the cluster, miR-92a was found to target *Tbr2* (Bian et al., 2013). Genesis of IPs was likewise found to be limited by miR-92b (Nowakowski et al., 2013). Conversely, miR-7 promotes IP genesis (Pollock et al., 2014). As noted above, miR-9^*^ and miR-124 target *Actl6a* (Baf53a) to promote BAF subunit switching, and are themselves repressed by Rest (Figure 12G; Son and Crabtree, 2014). Additionally, miR-9 and miR-124 target *Hdac5* for degradation (Figure 3H), and thus control neuritogenesis (Gu et al., 2018).

In the present analysis (Figure 12), three lncRNAs showed zonal expression restricted to the SVZ, and enrichment in *Tbr2*-GFP+ cells consistent with specific expression in bIPs: *A330008L17Rik* (log2FC = +2.40), *9630028B13Rik* (log_2_FC n.a.), and *A930024E05Rik* (log_2_FC = +1.97) (Figures 12A–C; Supplementary Table S2). An additional lncRNA, *Dubr* (log_2_FC = +1.77), was similarly expressed in SVZ/IZ, consistent with bIPs and new PNs (Figure 12D). *AI504432* (log_2_FC = +0.91), a lncRNA expressed specifically in bIPs with a high lateral gradient (Kawaguchi et al., 2008), was directly activated by Tbr2 and Tbr1. Similarly, lncRNA *4833418N02Rik* was significantly enriched in the Tbr2-GFP+ lineage, and was directly activated by Tbr1. Expression of lncRNA *Gm20735* was jointly activated by Tbr2 and Tbr1 (Figure 12G; Supplementary Table S4). Functions of these lncRNAs in cortical development are unknown, although some have been associated with different cortical neuron subtype fates, such as *A330008L17Rik* in PNs projecting axons to subcortical targets (Molyneaux et al., 2015).

Among miR genes, *Mir17hg* was highly enriched in *Tbr2*-GFP+ cells (log_2_FC = +1.96), and was localized in the inner VZ (Bian et al., 2013), suggesting that *Mir17hg* is specifically expressed by aIPs (Supplementary Table S2). Possibly, miR-17 expression in aIPs limits IP proliferation (Bian et al., 2013). *Mir9-2*, encoding miR-9/9^*^, was directly repressed by Tbr2 and Tbr1, suggesting that downregulation of these miRs may be important for PN differentiation (Figure 12G). In contrast, *Mir124a-1hg* (log_2_FC = +0.82) was highly expressed in new neurons of the IZ and CP (Figure 12E), suggesting it is necessary for neuron differentiation. One intriguing novel observation was a high rostral gradient of *Mir99ahg* in VZ/SVZ (Figure 12F). Significantly, miR-99 has been reported to target *Fgfr3* (Jiang et al., 2014), which is expressed in a high caudal gradient and regulates growth of occipitotemporal cortex (Hevner, 2005; Thomson et al., 2009). Thus, miR-99 may shape the *Fgfr3* gradient, and thereby regulate regional identity.

Together, these findings indicate that several lncRNAs are specifically expressed at high levels in IPs and new PNs, and that several miR genes are expressed with cellular or regional specificity. The gradient of *Mir99ahg*, and its possible targeting *Fgfr3*, suggest a new role for miR in cortical patterning. Finally, their direct regulation by Tbr2 and Tbr1 suggests that lncRNA and miR genes have significant functions in cortical development (Figure 12G).

### Neurodevelopmental processes controlled by EFs and regulated by Pax6, Tbr2, and Tbr1

The major findings from our analysis, summarized in Table 1, indicate that all kinds of EFs exhibit cell type-specific expression, and many EFs are regulated by Pax6, Tbr2, and/or Tbr1. These results implicate EFs in regulating cortical development at every stage of differentiation. Together with available functional information, our findings show that Pax6, Tbr2, and Tbr1 use transcriptional regulation of EF genes to modulate many important processes, notably IP genesis, laminar identity, and rostrocaudal regionalization of neocortex.

**Table 1:** Summary of differentiation-related EF gene expression and regulation by TFs.

**EF pathway/complex**	**Identity: identity-specific *genes***	**TFs and regulated genes**
DNA CpG methylation (repression)	RGP: *Dnmt1,-3a,-3b, Mbd2, Uhrf1*	Tbr2 represses *Dnmt3a, Mbd2*
DNA CpG demethylation (activation)	Caudal VZ/SVZ: *Gadd45g* PN lineage: *Tet1*	Pax6, Tbr2 repress *Gadd45g* Tbr1 activates *Tet1*
Histone acetylation (activation)	RGP: *Hat1, Kat7 (HBO1)*	Pax6 represses *Hat1*
	aIP and bIP: *Kat2a, Kat6b*	Tbr2 represses *Kat6b*
Histone deacetylation (repression)	aIP and bIP: *Hdac9*	Tbr2 represses *Hdac9*
	N-iz: *Hdac2, Mir9-2, Mir124a-1hg*	Tbr1, Tbr2 repress *Mir9-2*
	PN-iz: *Hdac5*	
	mixed: *Ankrd11*	Tbr1 activates *Ankrd11*
Trx H3K4 methylation (activation)	aIP and bIP: *Ash1l*	
	PN lineage: *Kmt2c*	Tbr1 activates *Kmt2c*
Trx H3K4 demethylation (repression)	N-vz: *Kdm1a*	Tbr2 activates *Kdm1a*
	N-svz: *Kdm5b*	Tbr1 activates *Kdm5b*
	Caudal VZ/SVZ: *Kdm5a*	
PRC2 H3K27 methylation (repression)	RGP: *Rbbp7, Aebp2*	
	PN lineage: *Rbbp4, Mtf2*	Tbr1 activates *Mtf2*
	Rostral VZ/SVZ: *Phf19* Caudal VZ/SVZ: *Suz12, Eed*	
PRC2 H3K27 demethylation (activation)	N-vz: *Kdm6b (Jmjd3)*	
	bIP: *Jarid2 (inhibits PRC2)*	Tbr2 activates *Jarid2*
	PN lineage: *Kdm6a (Utx)*	Pax6 represses *Kdm6a (Utx)*
PRC1 H2AK119 ubiquityl (repression)	RGP: *Pcgf5*	
	aIP and bIP: *Rybp (non-canonical)*	Tbr1 activates *Rybp*
	N-cp: *Pcgf3, Auts2 (non-canonical)*	Pax6, Tbr1 activate *Auts2*
	Caudal VZ/SVZ: *Cbx2*	
Other histone methylation or demethylation	aIP and bIP: *Kdm4c (GASC1)* PN-iz: *Setd6*	
	PN lineage: *Kdm7a*	Tbr2 represses *Kdm7a*
	N-iz: *Mllt3 (Af9)*	Pax6 activates *Mllt3*
ISWI chromatin remodeling	RGP: *Bptf, Rbbp7 (NuRF)*	
	aIP and bIP: *Baz2a,-2b (NoRC)*	Tbr2, Tbr1 repress *Baz2b*
	PN lineage: *Smarca5 (NoRC)*	
INO80 chromatin remodeling	RGP: *Ino80b* (INO80) PN lineage: *Srcap, Ep400, Kat5*	
CHD chromatin remodeling	RGP: *Ssrp1* (FACT), *Mbd2* (NuRD)	Tbr2 represses *Mbd2*
	aIP, caudal VZ: *Chd7*	Pax6, Tbr2 repress *Chd7*
	N-iz: *Chd3* (NuRD), *Hdac2* (NuRD)	Tbr2/Tbr1 activate *Chd3*
	PN lineage: Ctbp2 (NuRD related)	Tbr2, Tbr1 activate *Ctbp2*
	mixed: *Chd1* (FACT)	Pax6 represses *Chd1*
BAF chromatin remodeling	RGP: *Bcl7c*	
	N-vz: *Arid1b, Smarcd3, Bcl7a*	Tbr2 activ. *Smarcd3, Bcl7a*; Tbr1 activates *Arid1b*
	N-iz: *Actl6b, Bcl11b*	Tbr2, Tbr1 activate *Bcl11b*
	N-iz, caudal IZ/CP: *Bcl11a*	Pax6 activates *Bcl11a*
	PN-cp: *Brd9*	
	PN-cp, rostral CP: *Smarca2*	Pax6 activates *Smarca2*
	mixed: *Dpf3*	Tbr2, Tbr1 activate *Dpf3*
Rest and CoRest complexes (repression)	RGP: *Rest*	
	aIP and bIP: *Insm1, Rcor2*	Pax6, Tbr2 repress *Insm1*
	N-vz: *Kdm1a* (LSD1)	Tbr2 activates *Kdm1a*
ncRNA	bIP: *AI504432,A330008L17Rik 9630028B13Rik, A930024E05Rik Dubr*	Tbr2, Tbr1 activate *AI504432*
	N-iz: *Mir124a-1hg*	Tbr2, Tbr1 repress *Mir9-2*
	N: *Mir9-2*	Tbr2, Tbr1 repress *Mir9-2*
	unknown: *Gm20735*	Tbr2/Tbr1 activate *Gm20735*
	Rostral VZ/SVZ: *Mir99ahg*	

#### Regulation of IP genesis

**Figure 13:**
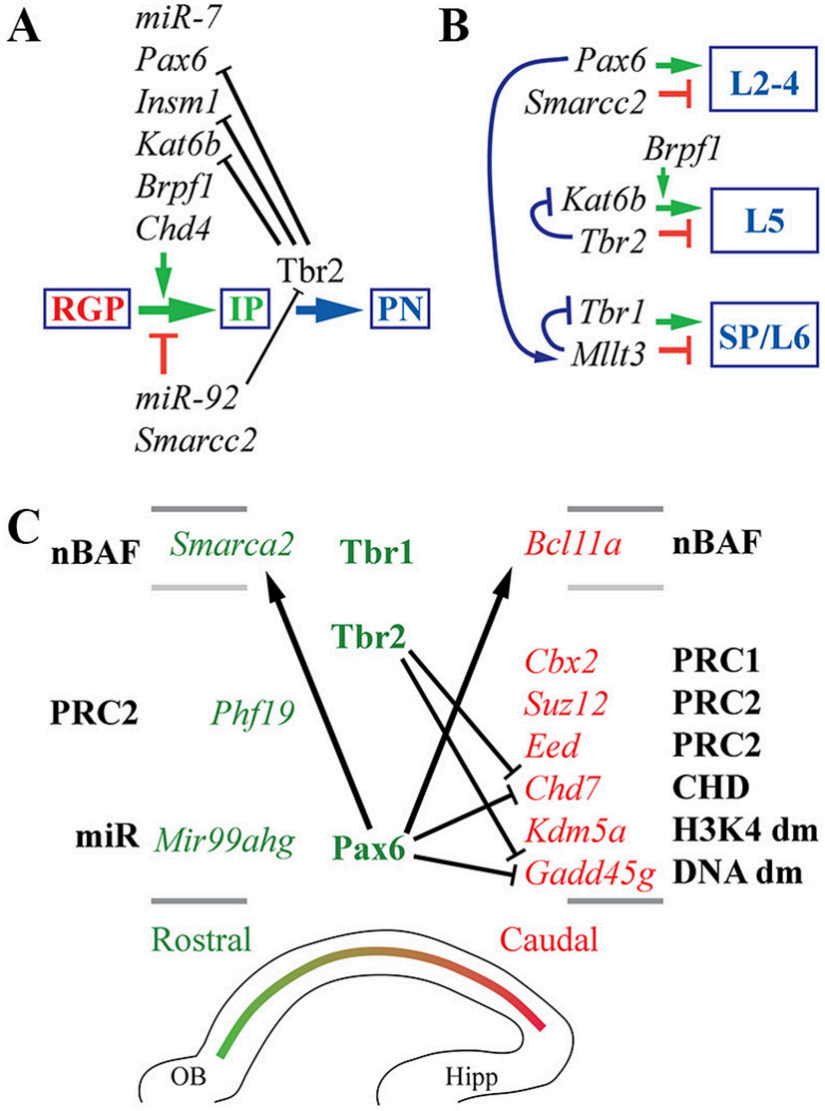
Cortex-specific neurodevelopmental processes regulated by EFs under the control of Pax6, Tbr2, and Tbr1. **(A)** IP genesis is reportedly regulated by multiple EFs, as well as TFs such as Pax6 and Insm1. Tbr2 directly represses IP-genic factors. Arrows: **(B)** Differentiation of all cortical layers is regulated by interacting EFs and TFs. SP: subplate. **(C)** Rostrocaudal regionalization is extensively regulated by TFs and EFs in an expansive gene regulatory network. Pax6 and Tbr2 regulate several regionally graded EF genes (italic), which are components of several different epigenetic complexes or systems (bold). Abbreviations: dm, demethylation; others as in text. Lines indicate zonal separation of IZ/CP above, and VZ/SVZ below. Arrows indicate direct transcriptional activation; bars, repression.

Previous studies have found that Pax6, Insm1, and Tbr2 each play distinct roles in IP genesis (Figure 13A). In *Pax6* null embryos, basal progenitors divide in the SVZ but do not express Tbr2, because Pax6 is required for *Tbr2* activation (Quinn et al., 2007). *Insm1* mutants exhibit severe reduction (~50%) of basal IPs with proportionately decreased *Tbr2* expression (Farkas et al., 2008).

In *Tbr2* cKO embryos, conflicting phenotypes have been reported. In studies using *Foxg1*-Cre recombinase, *Tbr2* inactivation caused ~75% reduction of basal IPs (Sessa et al., 2008). However, *Foxg1*-Cre heterozygosity itself causes ~38% IP deficiency (Siegenthaler et al., 2008), making *Foxg1*-Cre a sensitized, anomalous background. In contrast, *Tbr2* cKO mice produced with *Nes11*-Cre have normal or increased numbers of bIPs, which migrate into the IZ and divide ectopically (Mihalas et al., 2016). Importantly, *Nes11*-Cre is a transgene that does not interfere with cortical development. Thus, the data suggest that Insm1 and Pax6 promote IP genesis and differentiation, respectively; while Tbr2 promotes the transition from IP to PN, in part by repressing IP genes (Figure 11I).

Previously, many EFs have also been implicated in controlling IP genesis (Figure 13A). Among these, *Kat6b* (Morf, querkopf) was directly repressed by Tbr2 (Figure 3H; Supplementary Table S4). Morf (*Kat6b*) is a MYST family HAT that activates gene expression, and is required for forebrain growth (Thomas et al., 2000). It is unknown if IPs are reduced in *Kat6b* (Morf) deficient embryos, but deficiency of the MYST coactivator, Brpf1, has been found to reduce IP genesis and cortical growth (You et al., 2015). These findings indicate that Tbr2 is required to repress IP-genic EF (*Kat6b*) and TF (*Pax6, Insm1*) genes in IPs (Figure 13A).

#### Laminar fate

Previous studies have suggested that Pax6 promotes upper layer identity (Schuurmans et al., 2004); Tbr2 suppresses layer 5 identity (Mihalas et al., 2016); and Tbr1 promotes subplate and layer 6 identity (Hevner et al., 2001). Many EFs are also known to regulate laminar identity, and some are regulated by the Pax6→ Tbr2→ Tbr1 cascade (Figure 13B).

The present analysis found that Pax6 directly activated *Mllt3* (Af9), a YEATS domain acetylation reader that directly mediates *Tbr1* repression for upper layer identity (Büttner et al., 2010). Thus, Pax6 may promote upper layer identity in part by repressing lower layer identity. Paradoxically, Pax6 activates *Tbr1* indirectly (via *Tbr2*) to promote PN differentiation (Figure 11I), but also represses *Tbr1* indirectly (via *Mllt3*) to control laminar identity (Figure 13B).

Tbr2 may suppress layer 5 differentiation in part by directly repressing expression of *Kat6b* (Morf), a MYST family HAT that promotes layer 5 differentiation, as well as cortical growth (Thomas et al., 2000). In *Tbr2* cKO cortex, upregulation of *Kat6b* (log_2_FC = +0.18; *p* = 0.005) was associated with increased abundance of layer 5 neurons (Mihalas et al., 2016). The involvement of Morf (*Kat6b*) in layer 5 differentiation is supported by the phenotype of *Brpf1* mutant mice: Brpf1 is an activator of Morf (*Kat6b*), and *Brpf1* mutants have prominent layer 5 defects (You et al., 2015).

#### Rostrocaudal regionalization

The cerebral cortex is patterned by molecular expression gradients that confer different properties on cortical cells, according to their rostrocaudal and mediolateral coordinates (O'Leary et al., 2007). As part of this system, Pax6, Tbr2, and Tbr1 regulate molecular gradients at each stage of differentiation from RGPs→ IPs→ PNs (Bishop et al., 2000; Bedogni et al., 2010a; Elsen et al., 2013; Mihalas and Hevner, 2017). In the present study, many EFs that are expressed in rostrocaudal gradients were identified, including some that are directly regulated by Pax6 and Tbr2 (Figure 13C).

Both Pax6 and Tbr2 directly repressed two EF genes with high caudal gradients in VZ/SVZ: *Gadd45g* and *Chd7* (Figure 13C). These findings suggest that Pax6 and Tbr2 shape the *Gadd45g* and *Chd7* gradients. However, the roles of *Gadd45g* and *Chd7* in cortical regionalization remain unknown.

Interestingly, Pax6 directly activated the expression of BAF subunits *Smarca2* (Brm) and *Bcl11a* (Ctip1), in CP and IZ/CP respectively (Figure 13C). Since Pax6 is not expressed in IZ/CP, its ability to activate *Smarca2* and *Bcl11a* may depend on epigenetic mechanisms, such that Pax6 “unlocks” these genes in neurogenic progenitors, making them available for activation in PNs. The dependence of *Bcl11a*, a caudal enriched gene, on Pax6, a rostral enriched TF, suggests that while Pax6 may be necessary to unlock *Bcl11a*, Pax6 probably does not drive the *Bcl11a* gradient. While *Smarca2* has no known role in cortical regionalization, *Bcl11a* has been implicated in the acquisition of sensory cortex identity (Greig et al., 2016).

Although *Mir99ahg* was not directly regulated by Pax6→ Tbr2→ Tbr1, its high rostral expression gradient in the VZ (Figure 12F) was noteworthy because miR-99 targets *Fgfr3* (Jiang et al., 2014), which is expressed in a high caudal gradient and promotes growth of occipitotemporal cortex (Hevner, 2005; Thomson et al., 2009). Also, canonical PRC2 complexes play an important role in promoting occipital identity with high caudal gradients of *Suz12* and *Eed* (Figure 5J), but these PRC2 core genes were, in our analysis, not directly regulated by Pax6→ Tbr2→ Tbr1 (Figure 13C).

### Coordinate regulation of cortical development by TFs and EFs

The present study demonstrates that many types of EFs are direct targets of gene activation or repression by Pax6, Tbr2, or Tbr1 (Table 1). In many examples, the regulation of EFs by TFs was robust and affected multiple elements in an epigenetic system or signaling pathway. For example, Pax6, Tbr2, and Tbr1 activated multiple BAF subunit genes, to effect subunit switching and neuronal differentiation (Figure 10). In another example, Tbr1 activated non-canonical PRC1 subunits (*Rybp, Auts2*) in PNs (Figure 6). Also, many HATs and HDACs were regulated by this TF cascade (Figure 3). Overall, our results indicate that Pax6, Tbr2, and Tbr1 utilize EFs to modulate neurodevelopmental processes such as IP genesis, laminar fate acquisition, and regional identity (Figure 13). The Pax6→ Tbr2→ Tbr1 cascade itself emerges as a complex network with feedforward and feedback regulation (Figure 1B).

Epigenetic mechanisms appear well-suited to regulation of regional and laminar identity, persistent phenotypes that are initially determined in progenitor cells, then propagated into IPs and finally, new PNs. For example, the cortical “protomap” is initially specified in RGPs, then propagated into IPs and PNs, where regional identity continues to be refined (Bedogni et al., 2010a; Elsen et al., 2013; Alfano et al., 2014).

Besides EFs, other target genes regulated by Pax6, Tbr2, and Tbr1 can be identified using the same approach, and are currently under analysis. Through these studies, it will be possible to comprehensively profile gene expression by RGPs, IPs, and PNs; and to better understand how Pax6, Tbr2, and Tbr1 control the genesis of cortical PNs.

## Author contributions

GE designed and conducted experiments, produced new microarray data from Tbr1/2 mutant embryos, analyzed results, and wrote the manuscript. FB and RH produced microarray data. TB and JM analyzed data from microarray and ChIP-seq experiments. SL and JR produced and analyzed Pax6 ChIP-seq data. RH designed experiments, analyzed data, and wrote the manuscript.

### Conflict of interest statement

JR is cofounder, stockholder, and currently on the scientific board of Neurona, a company studying the potential therapeutic use of interneuron transplantation. The remaining authors declare that the research was conducted in the absence of any commercial or financial relationships that could be construed as a potential conflict of interest.
