# Supplementary figures and images for: The Epigenetic Factor Landscape of Developing Neocortex Is Regulated by Transcription Factors Pax6→ Tbr2→ Tbr1
Source: Front Neurosci. 2018 Aug 22;12:571. doi: 10.3389/fnins.2018.00571 (PMC6113890; doi:10.3389/fnins.2018.00571)

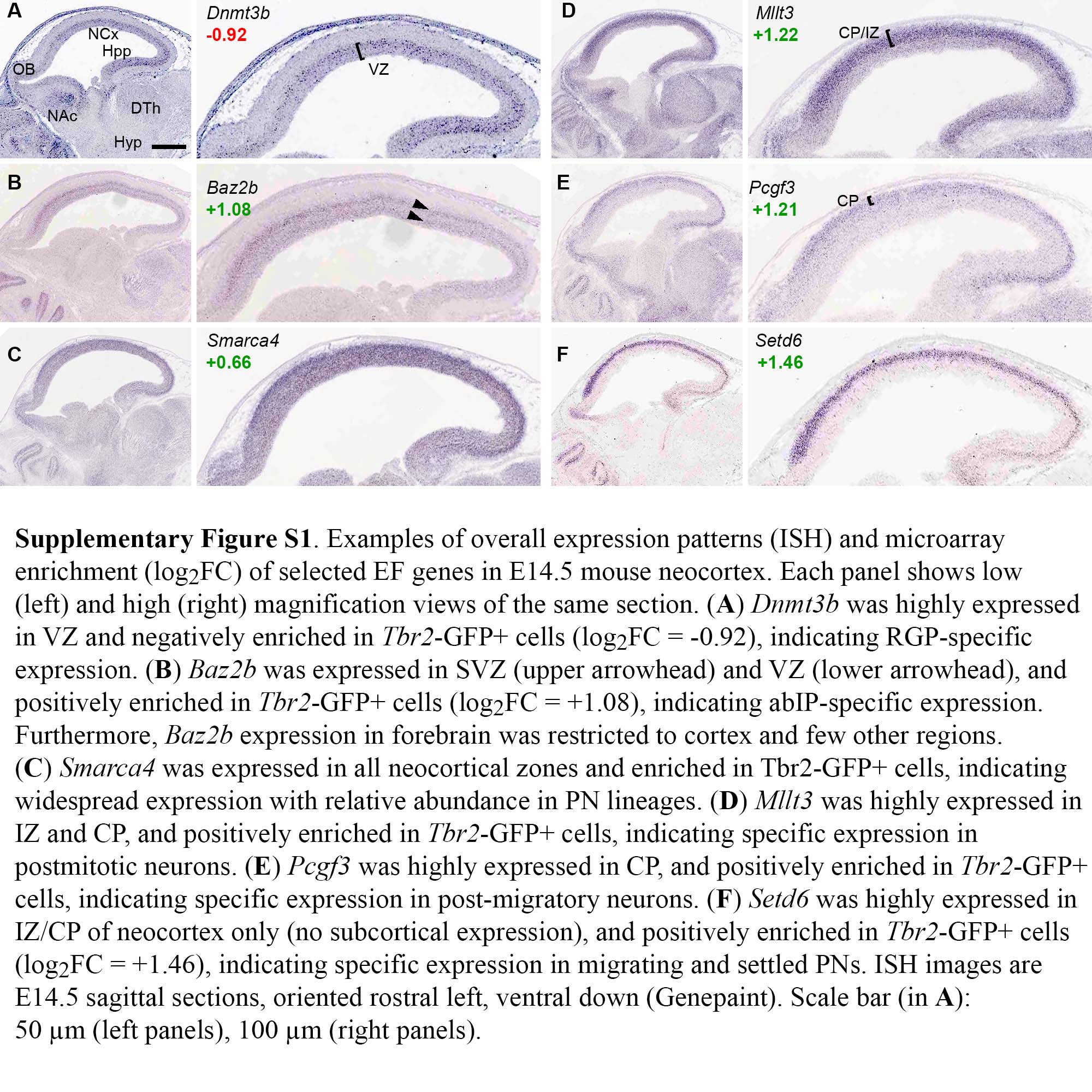

Supplement: Supplementary file 1 [file Image_1.jpeg]
