# Supplementary material for: The Epigenetic Factor Landscape of Developing Neocortex Is Regulated by Transcription Factors Pax6→ Tbr2→ Tbr1
Source: Front Neurosci. 2018 Aug 22;12:571. doi: 10.3389/fnins.2018.00571 (PMC6113890; doi:10.3389/fnins.2018.00571)
Supplement: Supplementary file 2 [file Table_1.docx]

**Supplementary Table S1. Microarray and ChIP-seq datasets used in the present study**

| **TF or mutant** | **Experiment** | **Source** | **Platform/Antibody** | **Reference** |
| --- | --- | --- | --- | --- |
| Pax6 | ChIP-seq | E12.5/13.5 cortex | anti-Pax6 (Millipore) | Pattabiraman et al. (2014) |
| *Pax6^Sey/Sey^* | Microarray | E15 cortex | Affymetrix MOE430A GeneChip | Holm et al. (2007) |
| Tbr2 | ChIP-seq | E14.5 cortex | anti-Tbr2 (Abcam ab23345) | Sessa et al. (2017) |
| *Tbr2* cKO (MA1) | Microarray | E14.5 cortex | Affymetrix Mouse Gene 1.0 | Elsen et al. (2013) |
| *Tbr2* cKO (MA2) | Microarray | E14.5 cortex | Affymetrix Mouse Exon ST 1.0 | present study |
| Tbr1 | ChIP-seq | E15.5 cortex | anti-TBR1 (Abcam ab31940) | Notwell et al. (2016) |
| *Tbr1* KO (MA1) | Microarray | E14.5 cortex | Affymetrix U430 2.0 | Bedogni et al. (2010) |
| *Tbr1* KO (MA2) | Microarray | E14.5 cortex | Affymetrix Mouse Exon ST 1.0 | present study |
| *Tbr1/2* KO/cKO (dKO) | Microarray | E14.5 cortex | Affymetrix Mouse Exon ST 1.0 | present study |
