# Supplementary material for: The Epigenetic Factor Landscape of Developing Neocortex Is Regulated by Transcription Factors Pax6→ Tbr2→ Tbr1
Source: Front Neurosci. 2018 Aug 22;12:571. doi: 10.3389/fnins.2018.00571 (PMC6113890; doi:10.3389/fnins.2018.00571)
Supplement: Supplementary file 3 [file Table_2.docx]

**Supplementary Table S2. EF genes expressed by specific cell types in developing neocortex**

| **Gene** | **Cell type** | **log_2_FC** | **ISH** | **EF type/complex** |
| --- | --- | --- | --- | --- |
| *Bcl7c* | RGP | -1.02 | n.a.* | npBAF |
| *Hat1* | RGP | -0.94 | VZ (outer) | HAT (type-B) |
| *Kat7* | RGP | -0.34 | VZ (outer) | HAT |
| *Ssrp1* | RGP | -0.51 | VZ | CHD1/FACT |
| *Ino80b* | RGP | -0.45 | n.a.* | INO80 (IES2) |
| *Dnmt3b* | RGP | -0.92 | VZ | DNA methyltransferase |
| *Dnmt3a* | RGP | -0.51 | VZ | DNA methyltransferase |
| *Uhrf1* | RGP | -0.87 | VZ | Histone reader |
| *Dnmt1* | RGP | -0.64 | VZ | DNA methyltransferase |
| *Bptf* | RGP | -0.76 | VZ (inner) | NuRF/ISWI |
| *Pcgf5* | RGP | -0.82 | n.d.* | PRC1-noncanonical |
| *Aebp2* | RGP | -0.47 | VZ (outer) | PRC2-noncanonical |
| *Rbbp7* | RGP | -0.69 | VZ | PRC2, NuRF, NuRD |
| *Rest* | RGP | -1.38 | VZ | REST/CoREST |
| *Chd7* | aIP | +0.80 | VZ (c>r) | CHD7 |
| *Mir17hg* | aIP | **+1.96** | VZ | miR |
| *Hdac9* | abIP | +0.60 | VZ, SVZ | HDAC-like (MITR) |
| *Kdm4c* | abIP | +1.72 | VZ, SVZ > CP | H3K9 demethylase |
| *Kat6b* | abIP | +1.24 | VZ, SVZ | HAT (MORF) |
| *Kat2a* | abIP | +0.68 | VZ, SVZ | HAT (GCN5) |
| *Baz2b* | abIP | +1.08 | SVZ > VZ | NoRC^?^/ISWI |
| *Baz2a* | abIP | +0.75 | SVZ > VZ, IZ | NoRC/ISWI |
| *Rybp* | abIP | +0.81 | SVZ,VZ > IZ, CP | PRC1-noncanonical |
| *Rcor2* | abIP | **+1.94** | SVZ, IZ > VZ | CoREST |
| *Ash1l* | abIP | +0.94 | VZ, SVZ >> CP | H3K36 methyltransferase |
| *AI504432* | bIP | +0.91 | SVZ > VZ (l>m) | lncRNA |
| *9630028B13Rik* | bIP | n.a.^#^ | SVZ | lncRNA |
| *A330008L17Rik* | bIP | **+2.40** | SVZ | lncRNA |
| *A930024E05Rik* | bIP | **+1.97** | SVZ | lncRNA |
| *Dubr* | bIP | +1.77 | SVZ, IZ | lncRNA |
| *Jarid2* | bIP | +0.88 | SVZ > CP | PRC2-noncanonical |
| *Smarca2* | PN-cp | +1.15 | CP | nBAF (Brm) |
| *Hdac5* | PN-cp/iz | +1.07 | IZ,CP | HDAC |
| *Setd6* | PN-iz | +1.46 | IZ | H2AZK7 methyltransferase |
| *Auts2* | N-cp | **+2.24** | CP >> VZ | PRC1-noncanonical |
| *Pcgf3* | N-cp | +1.21 | CP | PRC1-noncanonical |
| *Bcl11a* | N-iz | +1.50 | IZ, CP (c>r) | nBAF |
| *Bcl11b* | N-iz | +1.75 | CP > IZ | nBAF |
| *Actl6b* | N-iz | +0.67 | CP >> IZ | nBAF |
| *Mir124a-1hg* | N-iz | +0.82 | IZ, CP | miR |
| *Hdac2* | N-iz | +1.17 | CP,/IZ>VZ/SVZ | HDAC, NuRD, REST/CoREST |
| *Chd3* | N-iz | +0.76 | CP, IZ > VZ | NuRD |
| *Mllt3* | N-iz | +1.22 | IZ/CP > SVZ/VZ | Histone reader |
| *Dpf1* | N-svz | +0.68 | CP > SVZ | nBAF |
| *Kdm5b* | N-svz | +0.58 | CP, SVZ, IZ > VZ | H3K4me2/3 demethylase |
| *Smarcd3* | N-vz | +0.72 | CP >SVZ, IZ, VZ | BAF (BAF60c) |
| *Arid1b* | N-vz | +1.40 | CP > VZ | BAF-B (BAF250b) |
| *Ss18l1* | N-vz | +1.11 | VZ, SVZ > CP, IZ | nBAF (CREST) |
| *Bcl7a* | N-vz | +1.32 | CP > IZ, VZ | nBAF (BAF40a) |
| *Kdm6b* | N-vz | +1.28 | CP/IZ, SVZ > VZ | H3K27me3 demethylase (Jmjd3) |
| *Mtf2* | N-vz | +1.36 | CP, SVZ, IZ, VZ | PRC2 (noncanonical) |
| *Kdm1a* | N-vz | +1.14 | VZ, SVZ > CP, IZ | H3K4me1/2 demethylase (Lsd1) |

**Bcl7c*, *Ino80b*, and *Pcgf5* were enriched in microdissected VZ (Ayoub et al., 2011). ^#^*9630028B13Rik* was expressed in differentiating PNs on single-cell analysis (Telley et al., 2016). ^?^Baz2b complex is unknown, but homology to Baz2a suggests Baz2b might form a NoRC variant.
