# Supplementary material for: The Epigenetic Factor Landscape of Developing Neocortex Is Regulated by Transcription Factors Pax6→ Tbr2→ Tbr1
Source: Front Neurosci. 2018 Aug 22;12:571. doi: 10.3389/fnins.2018.00571 (PMC6113890; doi:10.3389/fnins.2018.00571)
Supplement: Supplementary file 5 [file Table_4.docx]

**Supplementary Table S4. EF and TF genes directly regulated by Pax6, Tbr2, and/or Tbr1 in developing neocortex**

| **Gene** | **Pax6 ChIP** | ***Pax6*  MA** | **Tbr2 ChIP** | ***Tbr2* MA1/2** | **Tbr1 ChIP** | ***Tbr1* MA1/2** | ***Tbr1/2*  MA2** |
| --- | --- | --- | --- | --- | --- | --- | --- |
| *Chd7* | + | +0.53 | + | +0.48/(+0.50) |  |  |  |
| *Gadd45g* | + | **+0.87** | + | +0.42/**+0.59** |  |  |  |
| *Auts2* | + | -0.39 |  |  | + | -1.42/n.a. |  |
| *Bcl11a* | + | -0.43 |  |  |  |  |  |
| *Mllt3* | + | -0.29 |  |  |  |  |  |
| *Smarca2* | + | -0.54 |  |  |  |  |  |
| *Hat1* | + | **+0.84** |  |  |  |  |  |
| *Chd1* | + | +0.34 |  |  |  |  |  |
| *Cbx4* | + | -0.29 |  |  |  |  |  |
| *Kdm6a* | + | +0.33 |  |  |  |  |  |
| *Baz2b* |  |  | + | (+0.12)/+0.30 | + | (-0.42)/0.23 |  |
| *AI504432* |  |  | + | -0.36/(-0.28) | + | **-2.20/-0.57** |  |
| *Bcl11b* |  |  | + | -0.38/-0.41 | + | -1.35/-0.41 |  |
| *Dpf3* |  |  | + | (-0.24)/-0.51 | + | (+0.08)/**-0.57** |  |
| *Mir9-2* |  |  | + | n.a./+0.15 | + | n.a./ 0.13 |  |
| *Ctbp2* |  |  | + | (-0.09)/-0.11 | + | -0.43/-0.09 |  |
| *Hdac9* |  |  | + | **+0.55/+0.68** |  |  |  |
| *Kat6b* |  |  | + | (+0.14)/+0.18 |  |  |  |
| *Jarid2* |  |  | + | -0.23/-0.20 |  |  |  |
| *Smarcd3* |  |  | + | -0.45/-0.58 |  |  |  |
| *Kdm1a* |  |  | + | (-0.05)/-0.12 |  |  |  |
| *Dnmt3a* |  |  | + | +0.25/+0.22 |  |  |  |
| *Bcl7a* |  |  | + | -0.25/-0.30 |  |  |  |
| *Kdm7a* |  |  | + | +0.25/ n.a. |  |  |  |
| *Gadd45a* |  |  | + | +0.45/(+0.10) |  |  |  |
| *Mbd2* |  |  | + | (-0.02)/+0.13 |  |  |  |
| *Rybp* |  |  |  |  | + | -1.47/(-0.06) |  |
| *Kdm5b* |  |  |  |  | + | -0.56/(-0.02) |  |
| *Arid1b* |  |  |  |  | + | -0.97/ n.a. |  |
| *Mtf2* |  |  |  |  | + | -1.53/(+0.04) |  |
| *4833418N02Rik* |  |  |  |  | + | -0.94/ n.a. |  |
| *Tet1* |  |  |  |  | + | -0.75/ n.a. |  |
| *Ankrd11* |  |  |  |  | + | -0.59/(-0.01) |  |
| *Kmt2c* |  |  |  |  | + | -1.09/ n.a. |  |
| *Chd3* |  |  | + | (-0.05)/(-0.21) | + | (-0.49)/(-0.20) | -0.32 |
| *Gm20735* |  |  | + | n.a./ (-0.23) | + | n.a. /(-0.23) | -0.47 |
| *Pax6* | + | **+1.20** | + | +0.36/+0.49 |  |  |  |
| *Tbr2* | + | **-1.07** | + | **-3.75/-3.74** |  |  |  |
| *Tbr1* |  |  | + | **-0.78**/-0.52 |  |  |  |
| *Insm1* | + | +0.55 | + | +0.46/0.36 |  |  |  |

TF binding (+) from ChIP-seq, and changes of gene expression in TF mutant cortex from microarrays, are indicated for genes where binding was present and the change in gene expression (log_2_FC) was significant. Values from both independent microarray experiments (MA1, MA2) are shown for *Tbr1* and *Tbr2* mutant cortex. All log_2_FC values were significant except those in parentheses, which are included for comparison between MA1 and MA2, or comparison to *Tbr1/2* MA. Values in **bold** were in the top 100 up- or down-regulated genes. n.a., not available (not probed on microarray platform).
